# Impact of Hedgehog modulators on signaling pathways in primary murine and human hepatocytes in vitro: insights into liver metabolism

**DOI:** 10.1007/s00204-024-03931-y

**Published:** 2024-12-23

**Authors:** Fritzi Ott, Christiane Körner, Knut Krohn, Janett Fischer, Georg Damm, Daniel Seehofer, Thomas Berg, Madlen Matz-Soja

**Affiliations:** 1 Faculty of Medicine, Rudolf Schönheimer Institute of Biochemistry, Leipzig University, Leipzig, Germany; 2 Division of Hepatology, Clinic and Polyclinic for Oncology, Gastroenterology, Hepatology, and Pneumology, University Hospital Leipzig, Leipzig, Germany; 3 Core Unit DNA-Technologies, Leipzig University, Leipzig, Germany; 4 Department of Hepatobiliary Surgery and Visceral Transplantation, University Hospital, Leipzig University, Leipzig, Germany; 5 Saxon Incubator for Clinical Translation (SIKT), Leipzig University, Leipzig, Germany

**Keywords:** Hedgehog signaling, Hepatocytes, Human, Murine, Modulator, Activator, Inhibitor

## Abstract

The Hedgehog (Hh) signaling pathway is essential for maintaining homeostasis during embryogenesis and in adult tissues. In the liver, dysregulation of this pathway often leads to liver cancer development. Recent studies also suggest that disturbances in the Hh pathway can affect liver metabolism in healthy livers through interactions with other signaling pathways, such as the Wnt/β-catenin pathway. As a result, the Hh pathway has emerged as a promising target for therapeutic intervention. However, little is known about the effects of Hh modulators on healthy hepatocytes. In our study, we investigated the effects of the Hh agonists SAG (300 nM) and triamcinolone acetonide (40 µM), as well as the antagonists RU-SKI 43 (100 nM), cyclopamine (5 µM), budesonide (25 µM), GANT61 (0.5 µM), and vismodegib (1 µM) on healthy mouse and human primary hepatocytes in vitro. We employed toxicological, transcriptomic, proteomic, and functional assays, including proliferation and Seahorse assays. Our results show that these compounds significantly impact metabolic pathways such as lipid and glucose metabolism at both transcriptional and protein levels. Mechanistically, our data suggest the involvement of both canonical and non-canonical Hedgehog pathways, a phenomenon not previously described in hepatocytes. These findings highlight the diverse effects of these compounds on signaling and key metabolic functions in the liver, which emphasizes the need to investigate the hepatic Hh cascade and its metabolic control in depth. As the compounds regulate different aspects of metabolism, they need to be carefully studied in appropriate model systems for specific therapeutic use.

**Supplementary Information:**

The online version contains supplementary material available at 10.1007/s00204-024-03931-y.

## Introduction

The Hedgehog (Hh) signaling pathway plays a crucial role in embryonic development by regulating tissue patterning, and in the adult it is essential for maintaining tissue homeostasis and activating regeneration processes. The pathway functions in canonical and non-canonical ways. In the canonical Hh pathway, Hh ligands such as Sonic, Indian or Desert Hedgehog (SHH, IHH, DHH) bind to the Patched (PTCH) receptor and release its inhibition of the Smoothened (SMO) receptor. This triggers the release of GLI family of zinc finger (GLI) transcription factors, which translocate to the nucleus to regulate the expression of genes controlling cell fate, growth and differentiation. In contrast, the non-canonical Hh pathway operates independently of GLI transcription factors and involves SMO-dependent but GLI-independent mechanisms that regulate processes such as cytoskeletal rearrangement, cell motility and metabolic reprogramming (Sup. Fig. S1) (Teperino et al. 2014).

Dysregulation of the Hh pathway, often due to mutations in PTCH, SMO or Suppressor of Fused (SUFU), or through ligand-dependent activation, has been implicated in several cancers, including hepatocellular carcinoma (HCC), where it is associated with increased tumor burden, invasion, metastasis and poor prognosis (Machado and Diehl 2018; Nguyen and Cho 2022).

Given the critical role of Hh signaling in liver disease, understanding the regulation of the hepatic signaling pathway in vitro and in vivo is imperative. It is poorly understood how changes in Hh signaling levels affects the vast network of metabolic pathways in the liver. Some data from transgenic mouse models provides a degree of insight, however there is a lack of in vitro models. The performance of selective Hh modulating compounds in hepatocytes remains unclear, but could be a valuable tool to investigate the specifics of the Hh pathway in this cell type.

To address this question, we screened several of the numerous Hh modulators targeting different levels of the pathway, two Hh activators (SAG, triamcinolone acetonide [TA]) and five inhibitors (cyclopamine [Cyclo], RU-SKI 43, budesonide [Bud], GANT61, and vismodegib [Vismo]). RU-SKI 43 prevents the palmitoylation of Hh ligands by targeting Hh acyltransferase (HHAT) (Petrova et al. 2013). Cyclo and Vismo inhibit Hh signaling while the agonist SAG activates Hh at SMO level (Taipale et al. 2000; Frank-Kamenetsky et al. 2002; Axelson et al. 2013). Bud and TA impair SMO translocation to the primary cilium, an organelle that is expressed only in a fraction of hepatocytes (Wang et al. 2012; Li et al. 2021), and GANT61 inhibits GLI transcription factors at the most downstream step of the pathway (Lauth et al. 2007).

The compounds were tested on murine and human primary hepatocytes, conducting toxicity and viability assays to ensure compound safety, followed by RNA sequencing, proteomic analyses, and functional assays to assess the molecular and metabolic changes induced by these modulators and gain insight into metabolic control of Hh signaling in the liver.

Our findings demonstrate that Hh modulators regulate the pathway in diverse ways, many of which differ from their known effects in other tissues and cell lines. In detail, some modulators show an activation of non-canonical Hh signaling. The metabolic consequences in hepatocytes are variable, as we observe effects in lipid, carbohydrate, xenobiotic and amino acid metabolism as well as cell cycle and autophagy. These broad effects can be attributed to the control that Hh exerts over expression of transcriptional regulators and should be carefully considered in future studies.

## Material and methods

### Maintenance and feeding of the mice

C57BL/6N mice were housed in a pathogen-free facility under a 12:12 h light–dark cycle according to European (Directive 2010/63/EU) and German guidelines for the care and safe use of experimental animals. All animal experiments were approved by the Landesdirektion Sachsen (permission numbers: T03/18, T09/22). The animals were fed ad libitum with regular chow (V1534-300 composed of 24.0 kJ% protein, 67 kJ% carbohydrate, 9 kJ% fat; usable energy: 13.5 kJ/g; ssniff® Spezialdiäten GmbH, Soest, Germany) and tap water throughout life. Male mice were euthanized at 12 weeks of age between 9 and 11 am after administration of an anesthetic consisting of ketamine, xylazine and atropine or an overdose of pentobarbital.

### Isolation and cell culture of primary murine and human hepatocytes

Primary hepatocytes from C57BL/6N mice were isolated using a previously described collagenase perfusion method or alternatively, using the Liver Perfusion Kit for GentleMACS OctoDisassociator (Miltenyi Biotech, Gladbach, Germany, 130–128-030) according to the manufacturer’s instructions (Gebhardt et al. 2003). The cell suspension was cleared of nonparenchymal cells using differential centrifugation (Matz-Soja et al. 2014). The pure hepatocyte fraction was either snap frozen for RNA isolation or cultured for 24 to 72 h with Hh inhibitors and activators according to a standard protocol (Kolbe et al. 2019). Stock solutions were prepared as followed: RU-SKI 43 (Tocris, Bristol, UK, 4886) 10 mM in DMSO, cyclopamine (Cyclo, Hycultec GmbH, Beutelsbach, Germany, HY-17024) 10 mM in DMSO, budesonide (Bud, Selleckchem, Cologne, Germany, S1286) 25 mM in DMSO, GANT61 (Selleckchem, S8075) 5 mM in ethanol, vismodegib (Vismo, Hölzel Diagnostika, Cologne, Germany, A10258) 50 mM in DMSO, SAG (Sigma-Aldrich, St. Louis, USA, SML1314) 1.2 mM in water and triamcinolone acetonide (TA, Selleckchem, S1628) 25 mM in DMSO. Final amount of DMSO and ethanol in incubation media was 0.5%.

Primary human hepatocytes from patients with benign liver diseases classified as healthy liver tissue were kindly provided by the group of Dr. Georg Damm/Prof. Seehofer at SIKT (Sächsischer Inkubator für Klinische Translation) Leipzig. Hepatocytes were isolated with a two-step collagenase perfusion as described previously (Pfeiffer et al. 2015; Damm et al. 2019).

### Ethics approval

The mice used in this study were maintained according to European (Directive 2010/63/EU) and German guidelines for the care and safe use of experimental animals. The animal experiments were approved by the Landesdirektion Sachsen (permission numbers: T03/18; T09/22). For human hepatocytes: All patients gave their informed consent according to the ethical guidelines of the Medical Faculty of Leipzig University (Ethical vote: registration number 322/17-ek, date 2020/06/10 ratified on 2021/11/30 and registration number 422/21-ek, date 2021/11/10).

### RNA sequencing

RNA was isolated using innuSOLV RNA Reagent (analytikjena, Jena, Germany, 845-SB-2090100) according to the manufacturer’s instructions. An additional purification step was performed after isopropanol precipitation of RNA. The pellet was reconstituted in 500 µl nuclease-free water and RNA was precipitated overnight with 3 M sodium acetate (pH 5.2) and 2-propanol.

RIN values of RNA were measured at Leipzig Core Facility using the Agilent Fragment Analyser (Agilent) according to the manufacturer’s instructions. RNA from three mice was pooled. 50 ng of total RNA were depleted of ribosomal RNA using the NEBNext® rRNA Depletion Kit v2 (NEB) according to the instructions of the manufacturer. Depleted RNA was transcribed using SuperScript IV reverse transcriptase (ThermoFisher) for 2 h at 55 °C. After second strand synthesis (TargetAmp kit (Epicentre)) the DNA was tagmented using the Illumina Tagment DNA TDE1 Enzyme and Buffer Kits, which fragments DNA and inserts partial sequencing adapter (Nextera) sequences. Final PCR amplification of the libraries was done using KAPA HiFi HotStart Library Amplification Kit with unique dual indexing by IDT® for Illumina Nextera DNA Unique Dual Indexes Sets. The barcoded libraries were purified and quantified using Qubit Fluorometric Quantification (ThermoFischer Scientific). Size distribution of the library DNA was analyzed using the FragmentAnalyzer (Agilent). Sequencing of 2 × 150 bp was performed with a NovaSeq sequencer (Illumina) according to the instructions of the manufacturer. After demultiplexing with bcl2fastq software (Illumina, v2.20) and polishing using fastp only sequences longer than 20 bp were further analysed. Reads were mapped against the human reference genome (hg38) using HISAT2 (Kim et al. 2015; Chen et al. 2018). Stringtie and the R package Ballgown were employed to calculate differential expression (Pertea et al. 2016). Expression data were normalized using the DESeq2 R bioconductor package (Love et al. 2014).

### Proteomics

For bottom-up LC–MS-based proteomics, sample preparation was conducted according to (Loroch et al. 2022). Tryptic peptides were separated via reversed phase using a 2 h acetonitrile gradient on a Neo Vanquish nanoLC online-coupled to an Exploris480 mass spectrometer (both from Thermo Scientific) operated in data-independent acquisition mode. Raw files were processed with Spectronaut v16.5 using the Mus musculus Swissprot database (17,152 sequences) and a mouse liver spectral library (Azimifar et al. 2014). Results were filtered at a 1% false discovery rate, only proteotypic peptides were considered and single hits were excluded.

### Statistics

Statistical analyses and chart design were performed using GraphPad Prism 9. Outliers were identified using the ROUT method with aggressiveness set to 1%. The cleaned data were used for subsequent statistical analyses and data depiction. A Principal Component Analysis (PCA) was done with R 4.2.2 and the package FactoMineR. The first two components are plotted. Data are plotted as mean values of biological replicates ± standard deviation. For statistical analyses of paired samples (control with corresponding incubations) paired t tests were performed. For the statistical analysis of viability assays and Vismo incubation paired two-way ANOVA were performed to analyze the influence of incubation time and concentration ranges. The null hypothesis was rejected at (corrected) p-values of * p < 0.05, ** p < 0.01, *** p < 0.001 and p **** < 0.0001.

Additional methods can be found in the supplementary material.

## Results

### Examination of toxic effects of selected compounds on hepatocyte viability

To test the effect on hepatocyte viability, we incubated primary murine hepatocytes with increasing concentrations of all seven modulators for up to 72 h and performed a viability assay every 24 h by quantifying adenosine triphosphate (ATP) levels relative to controls (Sup. Fig. S2). The results showed that cell viability remained stable during incubation with RU-SKI 43 and SAG over the tested concentration range for 72 h. In contrast, concentrations of Cyclo and GANT61 above 10 µM led to a rapid decrease in cell viability. For the glucocorticoids Bud and TA, viability was stable up to 50 µM. Hepatocyte incubation with Vismo showed stable viability over 72 h when exposed to lower concentrations up to 10 µM. This was also observed in human hepatocytes tested with Vismo alone. From these results we identified non-toxic concentrations of each compound and selected the following working concentrations for use in subsequent assays: RU-SKI 43 – 100 nM; Cyclo – 5 µM; Bud – 25 µM; GANT61—0.5 µM; SAG – 300 nM; TA – 40 µM for 48 h; and Vismo – 0.1/1/10 µM. The selected concentrations were compared with literature references (Sup. Table S1).

### Distinct regulatory effects of substances on gene and protein expression in murine hepatocytes

**Fig. 1:**
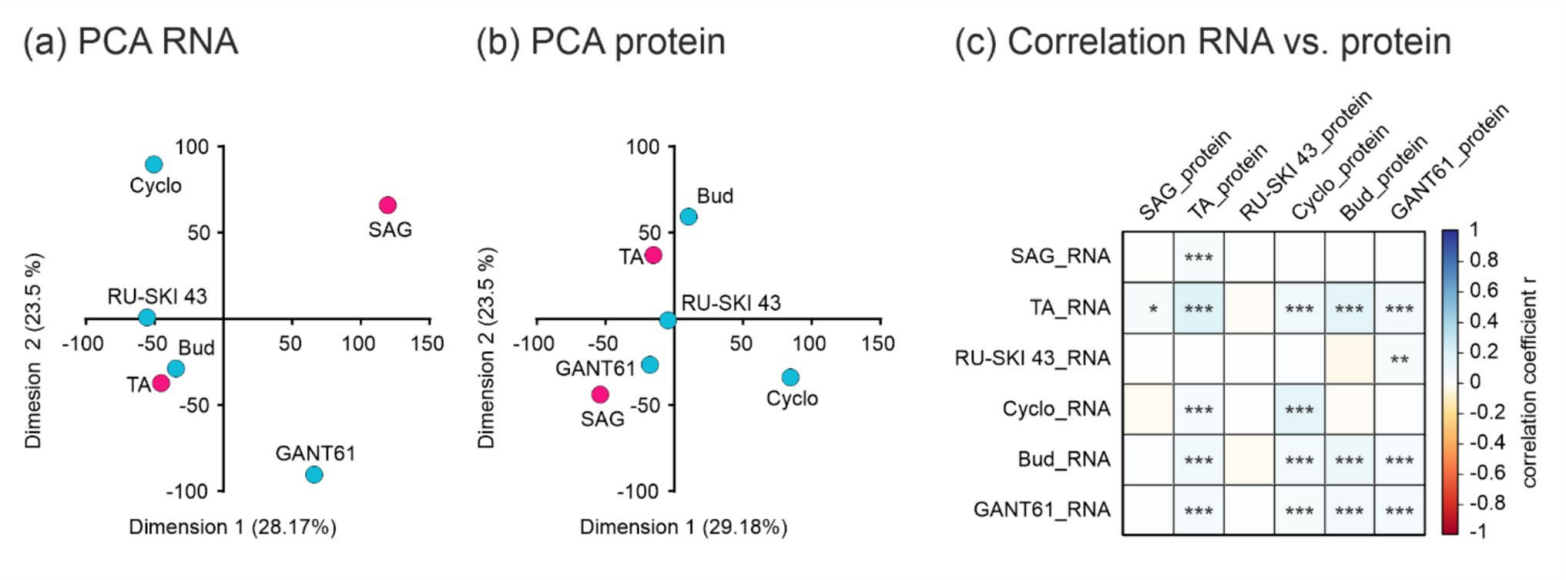
Principal component analysis (PCA) of **a** RNA and **b** Protein data. Hh activators (pink) and Hh inhibitors (blue) are plotted on dimensions 1 and 2. **c** Correlation analysis of RNA vs. protein data. p-values * ≤ 0.05; ** ≤ 0.01; *** ≤ 0.001

To determine the effects of selected compounds at the transcriptional and translational levels, we performed RNA sequencing (RNA-seq) analysis and proteomics on murine hepatocytes after 48 h of incubation. Transcriptome analysis revealed 15,000 differentially expressed genes and proteomics identified 5000 proteins. To directly compare these datasets, we mapped the corresponding genes to the identified proteins and performed principal component analysis (PCA) and correlation analysis. The PCA analyses reveal both similarities and differences in how the selected compounds affect gene and protein expression (Fig. 1a, b). TA and Bud are closely clustered in both the RNA and protein PCA, indicating that these substances have similar effects on both gene and protein expression. Cyclo shows a distinct expression pattern in both analyses, although the direction of differentiation is different. SAG shows a distinct gene expression pattern, but clusters more closely with GANT61 in the protein analysis, highlighting a complex regulatory mechanism. RU-SKI 43 shows some similarity to TA and Bud in RNA expression, but is more centrally located in the protein analysis, indicating a more average effect. GANT61 has a significantly different gene expression profile, but is more similar to SAG in the protein analysis. Calculated correlation coefficients for each compound showed that RNA and protein correlate positively for Bud, Cyclo and TA indicating consistency between RNA and protein expression. For RU-SKI 43 the result shows a moderate correlation, suggesting some level of consistency between RNA and protein expression (Fig. 1c). Despite the high statistical significance of the correlations, the actual correlation coefficients (r-values) are quite low, ranging from 0.193 for TA to −0.002 for SAG (Sup. Table S2). Taken together the PCA and correlation analysis the results show that substances like TA and Bud show high consistency across both transcriptional and translational levels, while others like SAG and GANT61 display more complex regulatory mechanisms. Despite the high statistical significance indicated by low p-values, the weak correlations (low r-values) suggest that factors beyond RNA expression significantly influence protein levels.

### Hh activators and inhibitors exert complementary pathway dynamics of Hh and Wnt signaling in vitro

**Fig. 2:**
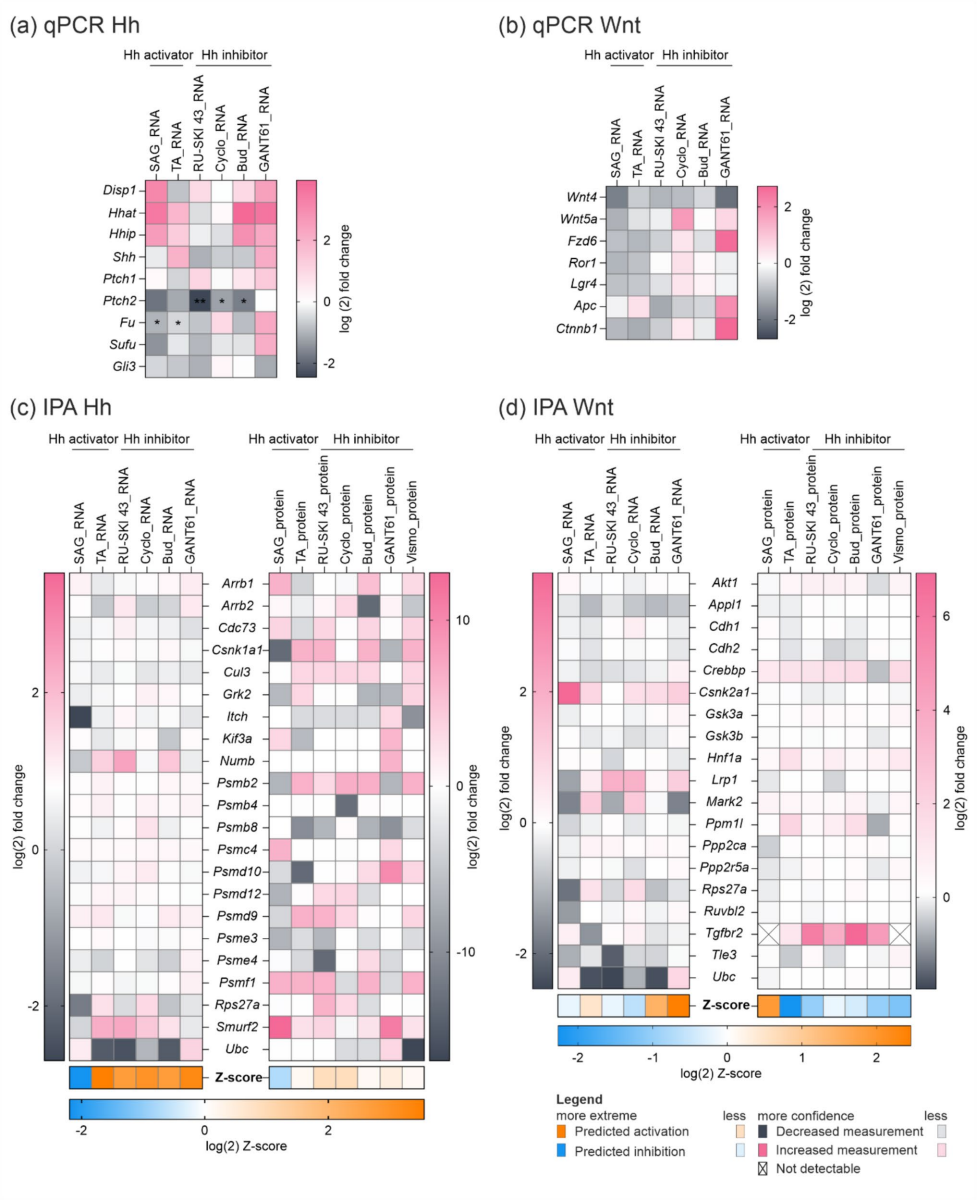
Effect of modulators on Hh and Wnt signaling pathway-related gene and protein expression in primary hepatocytes isolated from C57BL/6N mice. qPCR analysis of **a** Hh and **b** Wnt pathway-associated gene expression in primary hepatocytes incubated with Hh modulators for 48 h (left). mRNA expression is normalized to reference gene expression and shown as log(2) fold change. N = 3–4, n = 2. Paired t-test; p-values * ≤ 0.05, ** ≤ 0.01 (p-values are available in Sup. Table S3). IPA comparison analysis with integrated fold changes from RNA-seq and proteomic analysis for (**c**) ‘*Hh on state’* and **d** ‘*Wnt/β-catenin signaling’* pathways. Blocks indicate measured gene expression (pink/grey) or predicted regulation of gene expression and pathway outcome (orange/blue) for genes and proteins. A full list of gene and protein expression fold changes is available in Sup. Tables S4, S5. N = 3 (pooled), n = 1

After 48 h of incubation with the modulators, we assessed changes in the expression of key Hh signaling pathway genes using qPCR, RNA-seq, and proteomic analysis. qPCR revealed that RU-SKI 43 downregulated most Hh genes, including *Shh*, *Hhat*, *Disp2*, *Ptch2* (p = 0.0075), *Fu*, *Sufu*, and *Gli3*, indicating broad Hh pathway inhibition. Cyclo and Bud similarly affected *Shh* and *Ptch2* (p = 0.0486 and p = 0.0337), as well as *Sufu*, without altering *Gli3*. GANT61, a Gli1/2 inhibitor, downregulated only *Gli3*, while upstream molecules increased. Surprisingly, SAG and TA, despite being activators, showed expression profiles similar to Cyclo and Bud, downregulating *Ptch2*, *Smo*, *Sufu*, and *Gli3* (Fig. 2a). Vismo treatment in murine hepatocytes led to downregulation of Hh genes at 0.1 µM, with gene upregulation observed at 1 µM, and a shift from upregulation to downregulation at 10 µM between 48 and 72 h. In primary human hepatocytes, lower Vismo concentrations generally upregulated genes, though *GLI3* expression consistently increased (Sup. Fig. S3).

To explore regulation at transcriptional and protein levels, we analyzed RNA-seq and proteomics data using IPA software. Despite the limited detection of qPCR-identified genes in the RNA-seq data, the IPA analysis of the *'Hedgehog on state'* term revealed many regulated genes and proteins, including proteasome subunits and molecules involved in PTCH1 endocytosis and GLI activity, such as SMURF and CK1 (Shi et al. 2014; Yue et al. 2014). Z-score predictions suggested that the *'Hedgehog on state'* is generally activated by all compounds, except SAG, which is predicted to decrease this term in both gene and protein analyses (Fig. 2c; Sup. Fig. S4).

Considering the complementary roles of Hh and Wnt signaling, we also examined Wnt gene regulation following Hh modulation. qPCR showed that RU-SKI 43 and SAG downregulated all Wnt genes, indicating pathway inhibition. Cyclo treatment upregulated *Wnt5a*, *Fzd6*, and *Ctnnb1*, suggesting pathway activation, but also increased the inhibitory *Lgr4*. GANT61 strongly downregulated *Wnt4* and increased *Wnt5a*, while *Fzd6* and *Ctnnb1* increased, implying Wnt activation. However, *Apc*, part of the CTNNB1 destruction complex, was also upregulated. Bud and TA moderately regulated gene expression, with an overall downregulation of ligands and *Ctnnb1* (Fig. 2b).

Using RNA-seq and proteomic analysis, we observed similar detection limits for Wnt pathway genes as for the Hh pathway. Additional molecules were identified, including protein phosphatase subunits and adapter proteins such as APPL1 and APPL2. However, Z-score analysis indicated that Wnt signaling regulation in hepatocytes is less definitive compared to the Hh cascade, with RU-SKI 43 predicted to downregulate Wnt activity at both the gene and protein levels, while Bud showed a slight upregulation (Fig. 2d; Sup. Fig. S5).

In order to elucidate the mechanistic details of how Hh modulates metabolism, we performed ChIP-seq for GLI1 as well as ELISA experiments for regulatory proteins. ChIP-seq reveals binding sites of GLI1 in many genes involved in metabolism, such as *Srebf1* (lipid metabolism), *Cdk2* (cell cycle), *Mtor* (mTOR signaling), *Ctnnb1* (Wnt signaling) or *Sufu* (Hh signaling) as previously published (Sup. Table S9) (Ott et al. 2022). ELISA analysis of the regulatory proteins cyclin D1 (CCND1), peroxisome proliferator-activated receptor (PPAR) and pregnane X receptor (PXR) show an increase for Cyclo, statistically significant for CCND1, while expression levels for other modulators are barely affected (Sup. Fig. S8).

### Hh modulators show differential impacts on hepatocyte pathways and functions

**Fig. 3:**
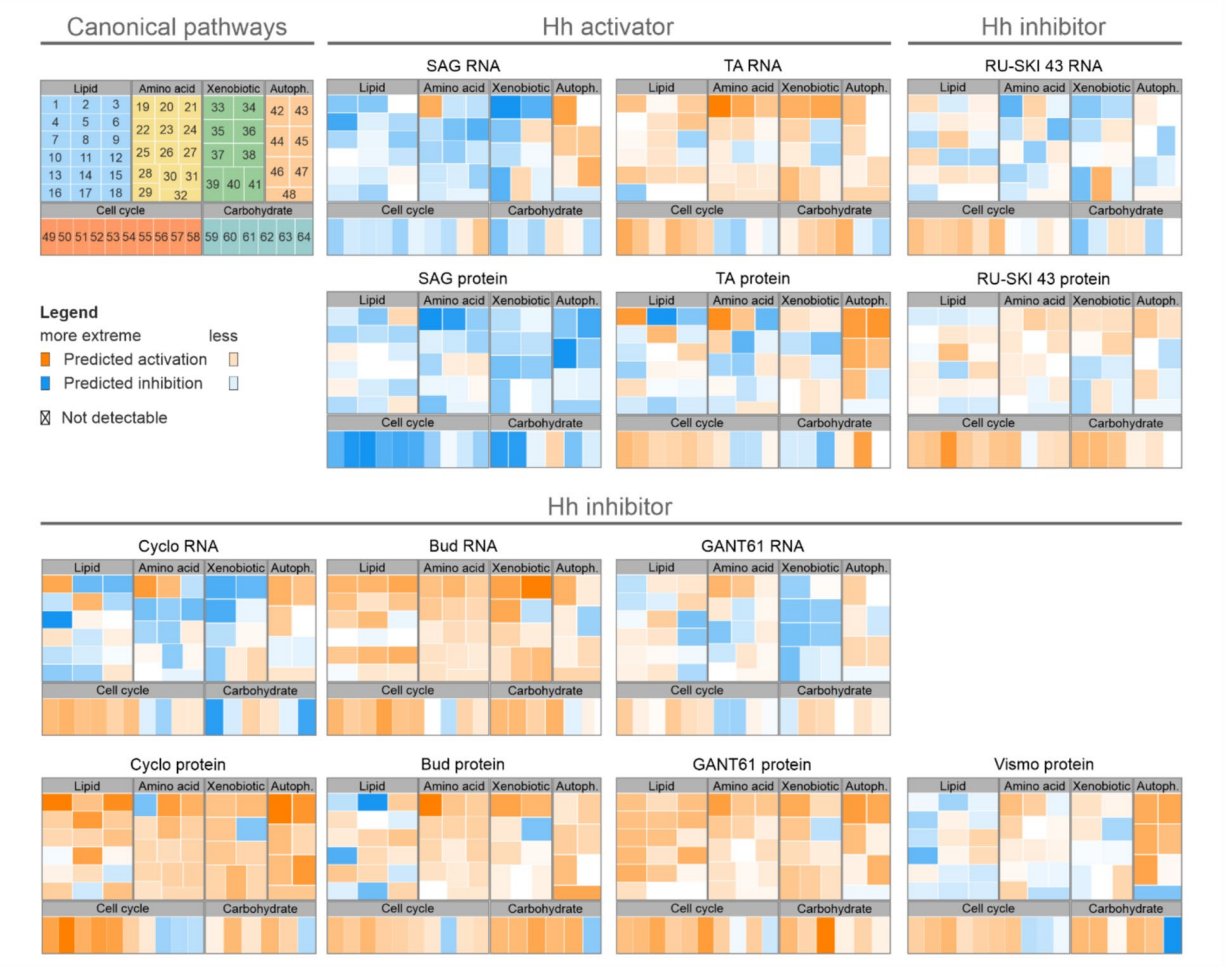
Canonical pathway regulation of primary murine hepatocytes. Regulation of canonical pathways of hepatocyte-relevant functions (Lipid, amino acid, xenobiotic, and carbohydrate metabolism, autophagy, cell cycle) is depicted as treemaps with positive (orange) or negative (blue) Z-score predicted by IPA based on transcriptomic and proteomic data. Canonical pathway annotation (pathway 1–64) is available in Sup. Fig. S7, log(2) Z-scores are available in Sup. Table S6

A multifactorial analysis using IPA matched regulated genes and proteins to pathways. After filtering results for hepatocyte-related functions – lipid, amino acid, xenobiotic, and carbohydrate metabolism, cell cycle, and autophagy – the results were displayed as treemaps with Z-scores (Fig. 3). For many canonical pathways, a clear difference between RNA and protein regulation can be observed.

The activators SAG and TA showed quite different regulations. While SAG generally downregulated pathways, except for autophagy gene expression, in the TA-treated hepatocytes, RNA expression was upregulated, with diverse protein regulation and increased autophagy and cell cycle proteins.

Among the inhibitors, RU-SKI 43 showed downregulation in lipid, amino acid, and xenobiotic-related pathways at the RNA level, while protein expression increased. Cyclo and GANT61 showed downregulation at the RNA level, but most protein pathways were upregulated. Bud treatment resulted in increased RNA and protein expression. RU-SKI 43 primarily upregulated cell cycle and carbohydrate metabolism proteins. Vismo increased protein expression, except for lipid and xenobiotic metabolism pathways.

For functional analyses of hepatocyte metabolism, we performed a metabolic assay using a Seahorse Analyzer to monitor cellular respiration by measuring oxygen consumption and extracellular pH. However, none of the modulators had a significant impact on respiratory parameters (Sup. Fig. S6a). In a steatotic assay, we analyzed the incorporation of lipids (palmitate and oleate; P/O) into hepatocytes after 48 h of Hh modulator treatment. Quantitative oil red staining did not detect any significant changes in lipid content (Sup. Fig. S6b). In a third assay, we analyzed the proliferative capacity of hepatocytes with or without hepatocyte growth factor (HGF) containing media. This was done by calculating the ratio of Ki67-positive nuclei to all nuclei stained by Hoechst. There were no significant changes observed, although there was a tendency for SAG to increase and Cyclo to decrease proliferative capacity (Sup. Fig. S6c).

## Discussion

Our study, using an integrative approach with cell viability assays, transcriptomic, proteomic, and functional analyses, revealed significant effects of specific Hh inhibitors and activators on healthy primary hepatocytes. We observed changes in both Hh and Wnt signaling pathways and in metabolic functions. Notably, most modulators did not perform consistently with expectations from the literature, highlighting the complexity of selecting appropriate compounds for hepatocyte studies. PCA of the omics data revealed unexpected clustering patterns, with Bud and TA clustering closely, underscoring their glucocorticoid role. Additionally, correlation analysis showed a weak but significant relationship between gene expression and protein translation, suggesting other factors influencing observed biological processes.

Hh and Wnt morphogens in the liver work together to maintain organ homeostasis and regulate metabolism (Kolbe et al. 2019). Hh activity negatively regulates Wnt through SFRP1, while Wnt activation affects Hh regulation via GLI3 (He et al. 2006; Alvarez-Medina et al. 2008). Repressing Hh signaling activates Wnt, whereas active Wnt signaling positively regulates Hh. However, the precise crosstalk mechanism in primary hepatocytes remains unclear. Our study found that Hh modulators had diverse effects on the mRNA expression of Hh and Wnt pathway components, as confirmed by qPCR and IPA analysis. We also examined the impact of these compounds on hepatic metabolism pathways.

**SAG**, typically recognized as an Hh activator, surprisingly decreased Hh activity in our hepatocyte models. This aligns with findings by Kwon et al. (2016) and Yao et al. (2023), who also observed no effect on *Smo* or *Ptch1* transcriptional activity in hepatocytes (Kwon et al. 2016; Yao et al. 2023). Regarding the Wnt cascade, our results were mixed; while Faria et al. (2019) found Wnt signaling induction after SAG treatment, our data showed downregulation at the gene expression level (Faria et al. 2019). Metabolically, SAG primarily downregulated pathways, except for slight increases in autophagy and some carbohydrate metabolism, and a decrease in maximal respiration. These results align with the metabolic reprogramming observed by Teperino et al. (2012) in adipocytes, where Hh signaling influences metabolism via the Smo-Camkk2/Lkb1-Ampk axis (Teperino et al. 2012). This non-canonical pathway might similarly affect hepatocyte metabolism, suggesting active canonical and non-canonical Hh pathways. Additionally, the study by Teperino et al. (2012) suggest that the metabolic profile of a cell can determine its responsiveness to Hh signaling, potentially influencing the balance between canonical and non-canonical pathways. This could explain the discrepancies between expected and observed gene expression changes in our hepatocyte experiments, highlighting the complex interplay between Hh signaling and cellular metabolism.

**TA**, a glucocorticoid, activates the Hh pathway by facilitating SMO movement into primary cilia (Wang et al. 2012). Our data showed similar regulation of Hh-related genes like *Shh* and *Hhip*, with IPA predicting an upregulated *'Hh on state'* at the RNA level, though no clear changes were seen in the proteomic data. This discrepancy may result from the small fraction of hepatocytes that can form primary cilia (Li et al. 2021). Unlike SAG, TA strongly downregulated Wnt signaling in proteomic data, likely due to its glucocorticoid function, known to inhibit Wnt cascade activity (Meszaros and Patocs 2020). Regarding metabolism, TA primarily influenced glucose metabolism and glycogen synthesis, consistent with glucocorticoid action, though its interaction with the Hh pathway remains speculative. As a mitogen, Hh regulates the cell cycle, with dysregulated activation promoting cancers like HCC (Agathocleous et al. 2007; Machado and Diehl 2018). TA uniquely leads to the upregulation of cell cycle-related pathways, fitting the expected regulation for Hh pathway prediction.

**RU-SKI 43**, known for inhibiting SHH ligand palmitoylation, surprisingly upregulated *Ptch2* expression, indicating Hh pathway activation rather than inhibition. This suggests that in primary hepatocytes, the canonical Hh pathway is more active than the SMO-independent non-canonical pathway. This activation might be related to RU-SKI 43’s cytotoxic effects, which some studies attribute to an unspecified "hedgehog-independent" function of HHAT (Rodgers et al. 2016). The Wnt signaling cascade was also activated, supporting the antagonistic crosstalk between Wnt and Hh pathways (Kolbe et al. 2019). RU-SKI 43 influenced metabolism in various ways, mostly upregulating pathways of amino acid and carbohydrate metabolism, as well as the cell cycle. It is described to affect the AKT and mTOR pathways, which engage in complex crosstalk with Wnt signaling via autophagy, potentially influencing Wnt beyond Hh modulation (Vadlakonda et al. 2013; Petrova et al. 2015)**.**

**Cyclo**, a naturally occurring SMO antagonist, showed moderate inhibitory effects on the Hh pathway in primary hepatocytes. Surprisingly, while qPCR data showed reduced expression of some Hh genes, IPA analysis indicated an upregulated *'Hh on state'*. This could result from the sensitivity of molecules included in the IPA term to the pathway's feedback loop. Cyclo treatment also upregulated PSMD9, associated with HCC progression, and downregulated PSME4, which activates mTOR signaling. Additionally, Cyclo’s ability to activate the non-canonical Hh pathway via the SMO-Ca2 + -AMPK axis might contribute to these findings, as similar effects have been observed in adipocytes (Teperino et al. 2012). Cyclo also influenced lipid metabolism, upregulating pathways through SREBF, a direct GLI1 target in hepatocytes (Ott et al. 2022).

**Bud**, another glucocorticoid, altered SMO localization, modulating Hh signaling by preventing SMO accumulation in primary cilia (Wang et al. 2012). While qPCR results showed inhibitory effects, IPA analysis predicted an upregulated 'Hh on state,' similar to RU-SKI 43 and Cyclo. Bud and TA showed different effects on gene and protein expressions, likely due to their distinct chemical structures and pharmacokinetics. Bud's impact on Wnt activity and hepatocyte metabolism was consistent with other glucocorticoids, though its interaction with the Hh pathway remains speculative (Xiao et al. 2014).

**GANT61**, a GLI1/GLI2 antagonist, upregulated most Hh pathway genes, with IPA predicting a similar *'Hh on state'*. The slight decrease in *Gli3* mRNA suggests pathway activation, as GLI3 acts as a repressor. GANT61 may not effectively inhibit GLI1 and GLI2 in this context. The downregulation of β-arrestin 2 suggests an interaction with primary cilia (Park et al. 2022). GANT61 also downregulated Wnt signaling components, consistent with previous reports in colorectal cancer (Si et al. 2022). Metabolically, GANT61 upregulated lipid metabolism and autophagy pathways, similar to Cyclo, supporting the hypothesis that it may also activate the non-canonical Hh pathway.

**Vismo**, an Hh pathway inhibitor used in treating BCC, showed high toxicity in clinical trials, with various adverse effects (Woltsche et al. 2019). Our study investigated Vismo's effect on Hh gene expression in murine and human hepatocytes. In murine hepatocytes, Vismo treatment produced concentration- and time-dependent inhibitory effects, while in human hepatocytes, it tended to upregulate *GLI1* and *GLI3* expression. This indicates Hh activation consistent with pathway regulation in literature using human fibroblasts, where the authors state that the activation in response to Vismo could be explained by a compensatory effect in the cells. Additionally, culture media composition can introduce bias as signaling pathways related to non-canonical Hh activation could be regulated (Eibenschutz et al. 2021). In general, studies have found that the effect of Hh-inhibitors seems to be more pronounced in cancerous tissue (Bhattacharya et al. 2008). The proteomic data supported these findings but did not provide a clear overall picture. Vismo's metabolic effects in hepatocytes were similar to those of Cyclo, suggesting a possible upregulation of the non-canonical Hh pathway, warranting further investigation in murine and human hepatocytes.

The exact mechanism of how Hh propagates effects on metabolism are likely as complex as the signaling pathway itself, however ChIP-seq data gives an insight. There are binding sites for GLI1 in many genes involved in metabolic control indicating that Hh signaling can directly affect lipid metabolism, cell cycle and Wnt signaling (Ott et al. 2022). *Sufu*, a negative regulator of Hh signaling, is a GLI1 target, showing a negative feedback loop that could attenuate Hh signaling. It is also worthy to note that *Akt1*, *Mtor* and *Rictor* are direct targets of GLI1, supporting crosstalk of Hh with the AKT-mTOR axis previously described in hepatocytes (Spormann et al. 2020). Another target of GLI1 is *Rxrb*, a nuclear receptor and binding partner to PPARs and liver X receptors (LXRs) (Zhan et al. 2012). This highlights the global effects that Hh modulation causes in hepatocytes and the difficulty of direct interventions in the metabolic network of the liver.

Protein levels of transcriptional regulators are slightly regulated in response to Hh modulation, leading to cell cycle activation with Cyclo. While in mice, PPAR factors are upregulated in response to Hh inactivation leading to a steatotic profile, there are little changes in our measured data, similarly with protein levels of PXR (Matz-Soja et al. 2016). However, the low abundancy of the proteins makes detection difficult and supports a comprehensive omics-based analysis to survey the network of metabolic pathways.

**Fig. 4:**
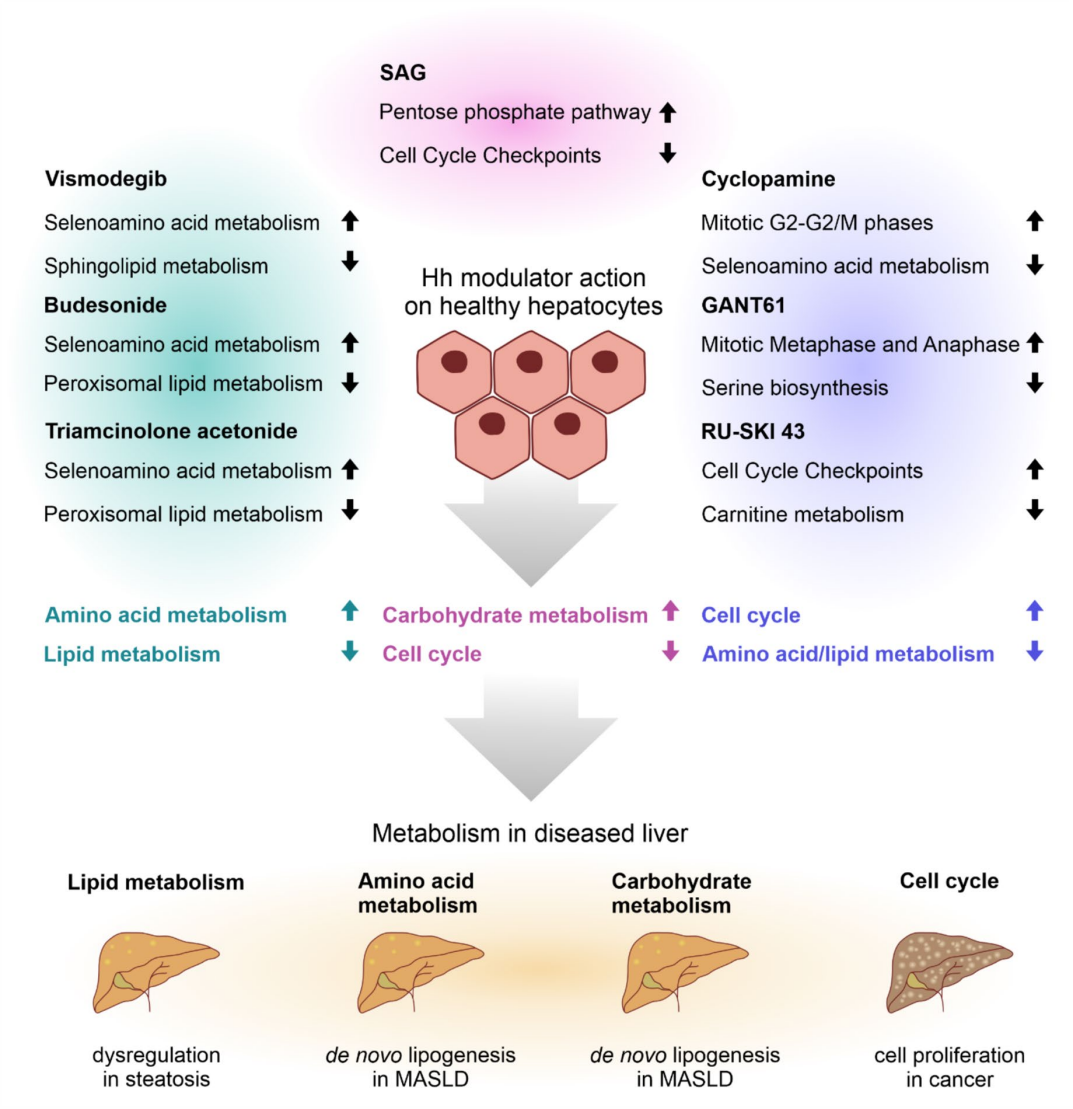
Summary of Hh modulator impact on hepatocytes. Hh modulator action on healthy hepatocytes was analyzed by Z-score (see Supplementary Tab. S6), with the most affected pathways shown here. Modulators were grouped based on similar effects on metabolism and examples of metabolic dysfunction in disease are depicted below

For a final overview, we grouped the screened Hh modulators according to the most regulated Z-scores in IPA (Fig. 4). This revealed clusters of modulators with similar effects on metabolism, namely Vismo/Bud/TA and RU-SKI 43/Cyclo/GANT61, while SAG remained isolated. Metabolic dysfunction is a hallmark of liver disease, with lipid metabolism being severely impaired, often leading to steatosis, amino acids and carbohydrates being utilized for further de novo lipogenesis in metabolic dysfunction–associated steatotic liver disease (MASLD), and activation of cell proliferation in liver tumors (Bisteau et al. 2014; Liao et al. 2024; Scoditti et al. 2024). This shows that the modulators cannot be ranked solely on the basis of their effects on Hh signaling, but that their impact on specific metabolic pathways needs to be considered in the clinical setting in order to target liver disease in an individualized manner.

In conclusion, based on our comprehensive study we observed that Hh signaling modulators significantly affect both Hh and Wnt pathways, as well as various metabolic processes in primary mouse and human hepatocytes. Interestingly, the expected inhibitory effects of certain compounds were not consistently reflected at the transcriptional and proteomic levels, highlighting the complexity of these pathways in liver cells. These findings underline the importance of thorough preclinical testing of Hh modulators, particularly with regard to off-target effects, potential unintended activation of non-canonical pathways and consequences for metabolic networks. Further research is essential to fully elucidate the specific molecular mechanisms, which may have significant implications for the development of safer and more effective therapeutic strategies especially for MASLD and cancer.

## Supplementary Information

Below is the link to the electronic supplementary material.

Supplementary file1 (DOCX 2194 KB)

Supplementary file2 (PDF 7580 KB)

Supplementary file3 (PDF 9949 KB)

Supplementary file4 (XLSX 49 KB)

## Data Availability

Raw data of RNA-seq and proteomic analysis were deposited with LiSyM SEEK (accessible at https://seek.lisym.org/data_files/696?code=nDZti3J1JoN5WVFH1acgT2DAefeShYgw1tznL%2BwE and https://seek.lisym.org/data_files/705?code=gE9Gw9AjgEYYj0Ow5CqP%2FfJN3k6POESucnd0SKQD).
