# Supplementary material for: Impact of Hedgehog modulators on signaling pathways in primary murine and human hepatocytes in vitro: insights into liver metabolism
Source: Arch Toxicol. 2024 Dec 23;99(3):1105–16. doi: 10.1007/s00204-024-03931-y (PMC11821798; doi:10.1007/s00204-024-03931-y)
Supplement: Supplementary file 1 — Supplementary file1 (DOCX 2194 KB) [file 204_2024_3931_MOESM1_ESM.docx]

Supplementary Material

**Article Title:** Impact of Hedgehog Modulators on Signaling Pathways in Primary Murine and Human Hepatocytes *in vitro*: Insights into Liver Metabolism

**Journal Name:** Archives of Toxicology

**Author Names:** Fritzi Ott, Christiane Körner, Knut Krohn, Janett Fischer, Georg Damm, Daniel Seehofer, Thomas Berg, Madlen Matz-Soja

# Corresponding author: Madlen Matz-Soja ([madlen.matz-soja@medizin.uni-leipzig.de](mailto:madlen.matz-soja@medizin.uni-leipzig.de))

# Supplementary Methods and Materials

## RNA isolation and quantitative real-time PCR (qPCR)

Total RNA was isolated using peqGOLD RNAPure™ (VWR International, Radnor, USA; 30-1010) and innuSOLV RNA Reagent (analytikjena, Jena, Germany) according to the manufacturer’s instructions. Reverse transcription was performed using a Proto-Script^®^ First Strand cDNA Synthesis Kit (New England Biolabs, E6300L) according to the manufacturer’s instructions. For qPCR analyses, gene-specific and intron-spanning primers were designed using Primer 3 software and were acquired from Microsynth (Balgach, Switzerland) unless otherwise noted (Sup. Tables S7, S8). A Biozym Blue S´Green qPCR Kit (Biozym, 331416XL) and a Rotor-Gene Q (Qiagen, Hilden, Germany) were used according to the manufacturer’s instructions. Expression levels were quantified in duplicate and calculated using internal amplification standards. Eukaryotic Translation Initiation Factor 3 Subunit F (*Eif3f*) and Peptidylprolyl isomerase A (*Ppia*) were used as reference genes for normalization in murine cells and β-actin (*Actb*) and *Ppia* in human cells.

## Seahorse Analyzer Cell Mito Stress Test

Primary hepatocytes were plated into XFe96 Cell Culture Microplates (Agilent, Santa Clara, USA, 103794-100) and cultured for 24 h. The Seahorse XF Cell Mito Stress Test (Agilent, Santa Clara, USA, 103010-100) was performed on the Seahorse XFe96 Analyzer (Agilent, Santa Clara, USA) according to manufacturer’s instructions with the concentrations: Oligomycin – 2 µM; FCCP – 1 µM, Rotenone/antimycin A – 0.5 µM. The data was normalized with confluence measured on the Celigo™ Imaging Cytometer (Celigo, Redwood City, USA) and analyzed using Seahorse Analytics (Agilent, Santa Clara, USA).

## Quantitative oilred staining

Primary hepatocytes were cultured for 48 h with cultivation media containing 120 mM palmitate (Sigma-Aldrich, P5585)/oleate (Sigma-Aldrich, O7501) 2:1 (P/O) versus methanol control (C). Cells were fixed using 4 % paraformaldehyde and washed thrice with ddH_2_O for 5 min, and with 60 % 2-propanol for 10 min. After drying completely, fat red 7B solution (Serva Electrophoresis, Heidelberg, Germany, 21335) was added for 10 min and differentiated with 60 % 2-propanol. After two washes with ddH_2_O samples were dried and fat red 7B extracted with 100 % 2-propanol for 10 min. Absorption was measured at 500 nm with 100 % 2-propanol as blank. Data was normalized to protein by with the Pierce™ BCA Protein Assay Kit (Thermo Scientific, Waltham, USA, 23225) according to manufacturer’s instructions.

## Proliferation assay

Primary hepatocytes were cultured for 48 h with 50 ng/µl hepatocyte growth factor (+HGF) or medium control (-HGF). Cells were fixed using 4 % paraformaldehyde and permeabilized with 1x PBS + 0,5 % Tween-20. After blocking with 5 % BSA samples were incubated with Ki67 antibody (Abcam, Cambridge, United Kingdom, ab15580) overnight. Secondary antibody (Jackson Immunoresearch, West Grove, USA, 711-606-152) was incubated for an hour at RT with 5 µg/ml Hoechst 33342 (Biomol, Hamburg, Germany, ICT-639). Samples were mounted with mowiol containing 0.1 DABCO and imaged on Keyence BZ-X800 with 10x APO objective (Keyence, Osaka, Japan). Hoechst+ nuclei were counted automatically with BZ-X800 Analyzer software, Ki67+ nuclei were counted manually in ImageJ.

**Proteomics**

Proteomics Sample Preparation

For proteomics analysis, cells were harvested by scraping in presence of 1 % sodium dodecyl sulfate (w/v) including one tablet cOmplete Mini (Roche, Basel, Switzerland) per 10 ml. Samples were homogenized using 3x30 s of ultrasound, followed by centrifugation at 20,000 g at 4°C for 10 min. The intermediate phase was transferred to a new tube. Protein concentrations were determined using a bicinchoninic acid assay (Pierce, Thermo-Fisher Scientific, Bremen, Germany). Cysteines were reduced by adding dithiothreitol to a final concentration of 10 mM and incubation for 30 min at 56°C followed by alkylation of the free sulfhydryl groups by adding iodoacetamide to a final concentration of 20 mM and incubation for 30 min at RT in the dark. Samples were processed by filter-aided sample preparation in 96-well format (Loroch et al., 2022). Therefore, samples were diluted with 8 M urea in 150 mM TRIS-HCl, pH 8.5, to a final concentration of 0.25 % SDS and loaded onto the filters. The liquid was pushed through the membrane by positive pressure. The protein fraction retained on the filter was washed two times with 200 µl 8 M urea in 150 mM Tris-HCl, pH 8.5 and two times with 50 mM ammonium bicarbonate (ABC). Digestion was performed in 100 µl 50 mM ABC, 1 mM CaCl_2_ and trypsin (Promega, Sequencing Grade Modified, Madison, USA) was added in an enzyme-to-protein ratio of 1:32 (w/w). After incubation for 15 h at 37°C, the resulting peptide fraction was recovered by positive pressure and the filters were washed with 100 µl 50 mM ABC and 100 µl of water to recover residual peptides. Samples were acidified by the addition of trifluoroacetic acid (TFA) to a final concentration of 1 %.

LC-MS

Samples were analyzed using an Exploris 480 mass spectrometer (MS) online-coupled to a Vanquish Neo nanoLC (both from Thermo Scientific including columns, Dreieich, Germany) equipped with a PepMap Neo Trap precolumn (0.3 x 5 mm, 5 µm particles) and a DNV PepMap Neo main column (0.075 x 50 mm, 2 µm particles). Peptides were loaded onto the precolumn in 0.1 % TFA at 60 µl/min using a loading volume of 20 µl. Peptides were separated on the main column at a flow rate of 400 nl/min using a stepped linear gradient from 1 to 18 % acetonitrile (ACN) in 100 min and from 18 to 29 % ACN in 20 min in presence of 0.1 % formic acid. The MS was operated in data-independent acquisition (DIA), using a survey with a resolution of 60,000 followed by 40 MS/MS scans spanning the mass range of 430-670 m/z using a resolution of 30,000 and an isolation width of 6 m/z. The normalized collision energy was set to 32 %, the maximum AGC target values were set to 300 and 2250 % for MS and MS/MS scans, respectively. The ion injection times were set to auto and the polysiloxane signal at m/z 445.12003 was used as internal calibrant.

Data analysis

All DIA runs were analyzed with Spectronaut v16.5 (Biognosys AG, Zurich, Switzerland) using BGS factory settings, but considering only proteotypic peptides and using QUANT2.0 for quantification. For identification, a database search was conducted against all reviewed *Mus musculus* entries from Uniprot (www.uniprot.org, 11-2023, 17,152 target sequences) and a spectral library was generated from DDA runs from mouse liver cells (Azimifar et al., 2014) (replicates 1+2 of 2D-LC-MS mouse liver cell analysis) downloaded via the identifier PXD000867 from ProteomeXchange (www.proteomexchange.org, (Deutsch et al., 2023)).

**Chromatin immunoprecipitation with sequencing (ChIP-seq)**

Hepatocytes from four male C57BL/6N mice were pooled and used for ChIP-seq performed by the company Active Motif (Carlsbad, CA, USA) as described previously (Ott et al., 2022).

**Enzyme-linked immunosorbent assay (ELISA)**

For ELISA experiments, samples were lysed with Homogenisator, Precellys® 24 (VWR International, Radnor, USA) in RIPA buffer and centrifuged at 13000xg, 4 °C for 5 min. Protein concentration of supernatant was measured with the Pierce™ BCA Protein Assay Kit (Thermo Scientific, Waltham, USA, 23225). ELISAs were performed according to the manufacturer’s instructions with the following kits: Mouse Cyclin D1 ELISA Kit (A1797), Mouse PXR ELISA Kit (A270943) and Mouse PPAR alpha ELISA Kit (A2523) (Antibodies.com Europe AB, Stockholm, Sweden).

## IPA analysis

RNA-seq as well as proteomic data were analyzed with Ingenuity Pathway Analysis (IPA) by Qiagen (QIAGEN IPA by QIAGEN Inc., <https://digitalinsights.qiagen.com/IPA>). A comparison analysis with an expression fold change cutoff of ≤ - 2.0 and ≥ 2.0 was conducted. A p-value cutoff of 1.3 was chosen for further analysis. An additional Z-score cutoff of 1.5 was used to depict pathways affected by Hh modulation.

## Figures

The figures were made with CorelDRAW®2020 (Version 22.0.0.412; © 2020 Corel Corporation).

# Supplementary Figures and Tables

## Supplementary Figures

**Sup. Fig. S1** Overview of the canonical and non-canonical Hh signaling pathways and regulatory compounds used in the study. (a) When inactive, smoothened (SMO) is inhibited by patched (PTCH) receptors. Suppressor of fused homolog (SUFU) sequesters GLI family zinc finger (GLI) proteins, making them available for phosphorylation by casein kinase 1 (CK1), protein kinase A (PKA), and glycogen synthase kinase 3β (GSK3β). GLI factors are processed to their inhibitory forms, suppressing target gene transcription. (b) When active, ligands sonic, indian, and desert Hh (SHH, IHH, DHH) bind to PTCH, causing its exit from the ciliary membrane and releasing SMO inhibition. SMO and SUFU are transported in primary cilia by kinesin family member 7 (KIF7), releasing GLI factors, which move into the nucleus as activators to start transcription (Petrova and Joyner, 2014; Wu et al., 2017). The Hh modulators investigated are shown with their action sites. Non-canonical signaling is either cilia-dependent (c), promoting Warburg metabolism via AMP-activated protein kinase (AMPK); or cilia-independent (d), resulting in apoptosis via PTCH receptor or cytoskeletal rearrangement and cell migration via Ras Homolog Family Member A (RHOA), Rac family small GTPase 1 (RAC1) and SRC proto-oncogene, non-receptor tyrosine kinase (SRC) (Teperino et al., 2014). The figure was adapted from Wu et al. (2017) (Wu et al., 2017).


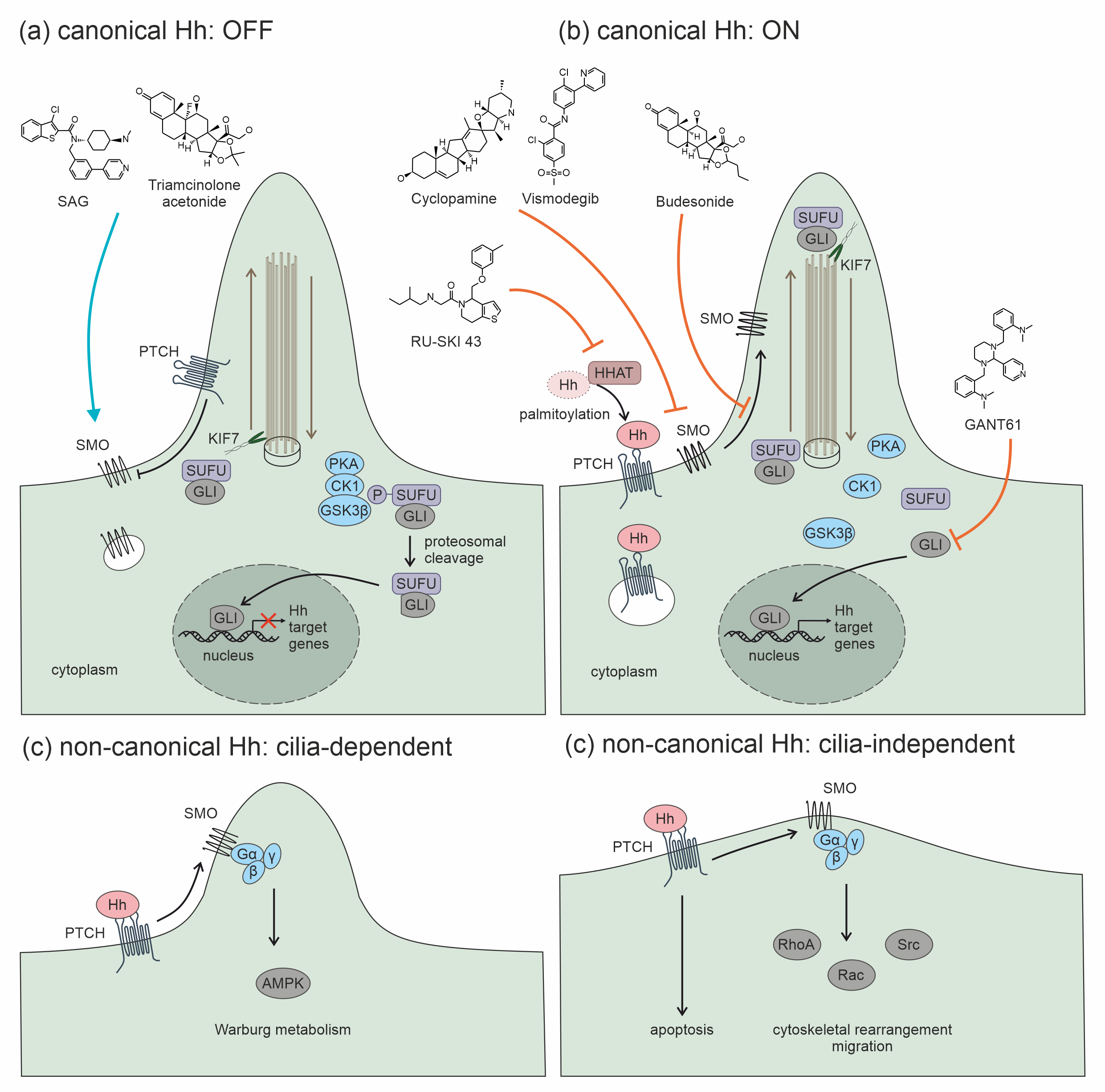


**Sup. Fig. S2** Determination of non-toxic concentrations of small molecule Hh pathway modulators. Viability of primary murine hepatocytes was tested with dilution series of (a): Hh activators, (b): Hh inhibitors in murine hepatocytes and (c): Vismo additionally in human hepatocytes over 72 h and is shown in relation to respective controls. Error bars show SD. N = 3, n = 3. Two-way ANOVA


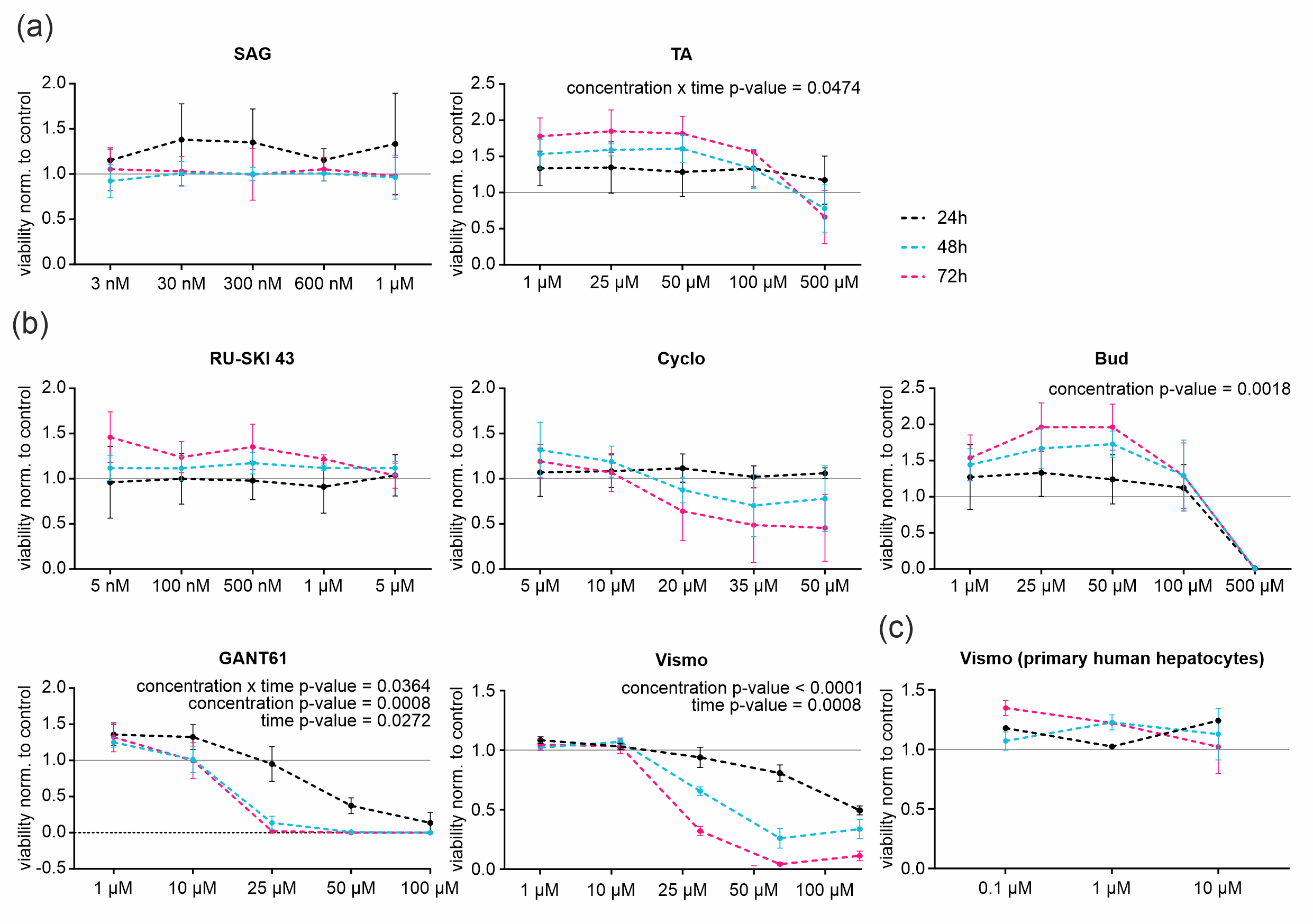


**Sup. Fig. S3** qPCR analysis of Hh pathway-associated genes in (a): primary murine hepatocytes (C57BL76N) and (b): primary human hepatocytes incubated with Vismo for 48 h and 72 h. mRNA expression is normalized to reference gene expression and depicted as log(2) fold change. Crossed squares indicate non-detectable values. N = 3-8, n = 2 (a); N = 1-3, n = 2 (b); Two-way ANOVA


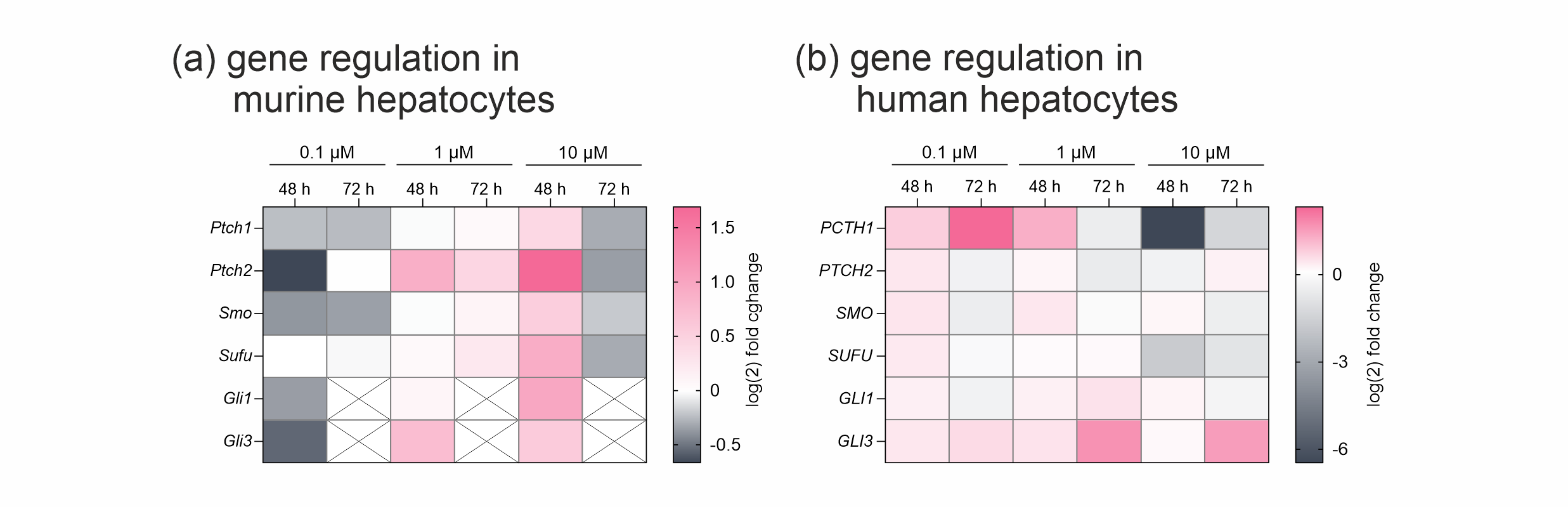


**Sup. Fig. S4** Interactive heat map of Hh signaling modulation in vitro. Depicted is the *‘Hh on state’* signaling pathway with increased (red) and decreased (green) measurement and predicted activation (orange) and inhibition (blue) of pathway components as well as predicted relationships. (The data is available in the PDF file *FigS4*)

**Sup. Fig. S5** Interactive heat map of Hh signaling modulation in vitro. Depicted is the ‘*Wnt/β-catenin signaling’* pathway with increased (red) and decreased (green) measurement and predicted activation (orange) and inhibition (blue) of pathway components as well as predicted relationships. (The data is available in the PDF file *FigS5*)

**
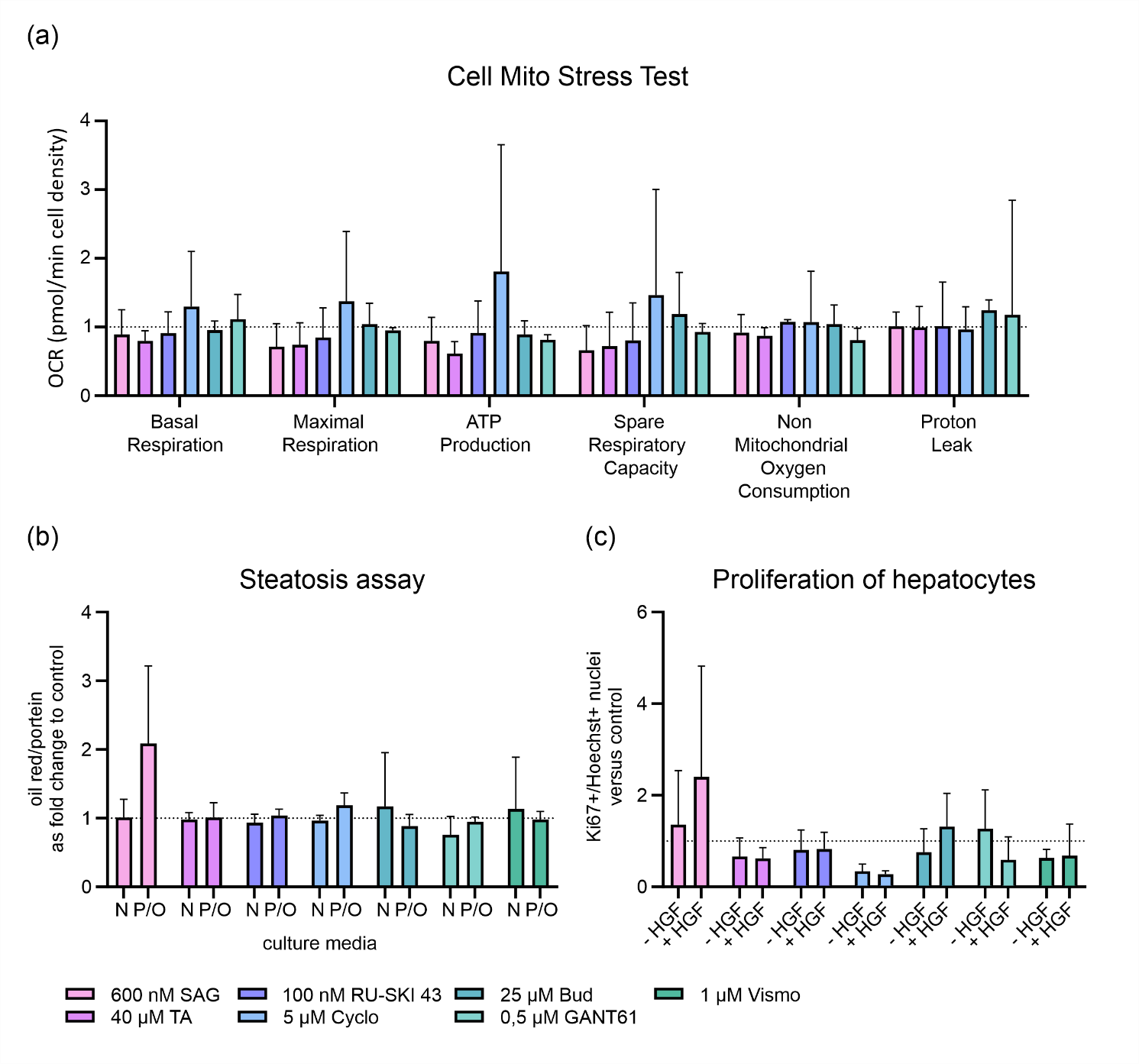
Sup. Fig. S6** Functional analyses of primary hepatocytes treated with Hh modulators. (A): Analysis of respiratory parameters in Seahorse Cell Mito Stress Test. N = 3-4, n = 5-6, One-way ANOVA. (B): Analysis of lipid incorporation after Hh modulator treatment with normal (N) or palmitate/oleate containing (P/O) media with quantitative oil-red staining. N = 4, n = 2, One-way ANOVA. (C): Analysis of proliferative capacity of hepatocytes after Hh modulator treatment with 40 ng/µl hepatocyte growth factor (+HGF) or normal culture media (-HGF). Hepatocyte nuclei were stained with Hoechst and anti Ki67 antibody and total versus proliferative nuclei were counted with Keyence and ImageJ. N = 3, n = 5, One-way ANOVA

**Sup. Fig. S7** Canonical pathway overview. The pathways regulated in transcriptomic and proteomic datasets are shown as a tree map according to general terms: Lipid metabolism, Amino acid metabolism, Xenobiotic metabolism, Autophagy, Cell cycle, and Carbohydrate metabolism.


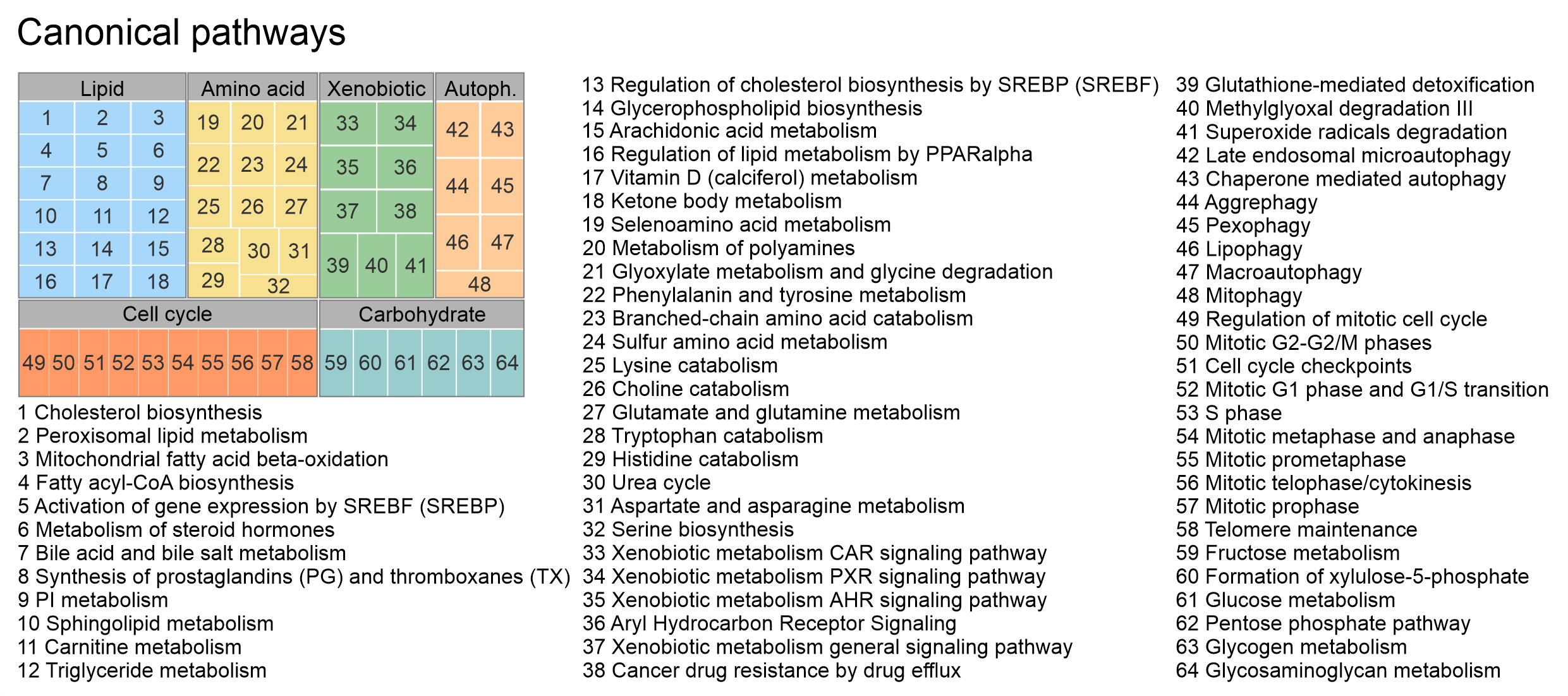


**
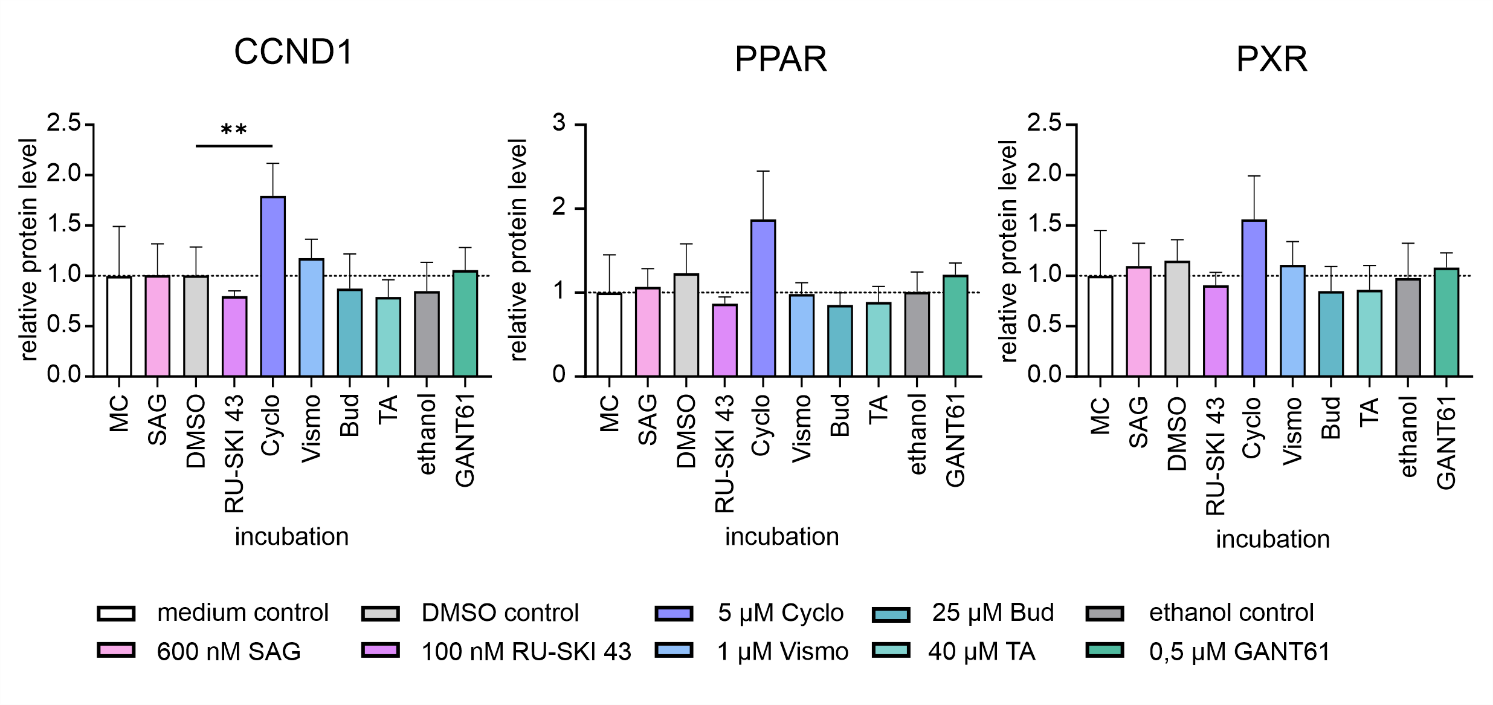
**

**Sup. Fig. S8** ELISA results for cyclin D1 (CCND1), peroxisome proliferator-activated receptor (PPAR) and pregnane X receptor (PXR). Optical density was measured at 450 nm and fitted to a standard curve. Data was normalized to protein concentration and analyzed in relation to the medium control. N = 3, n = 1, paired t-test and One-way ANOVA; p-values * ≤ 0.05

## Supplementary Tables

**Supplementary Table S1** Overview over concentrations of Hh activators and inhibitors used to modulate Hh signaling in primary murine hepatocytes and comparison to literature reports.

| Hh modulator | Concentration used  in hepatocytes | Concentrations used in literature | Cell type used in literature | Reference |
| --- | --- | --- | --- | --- |
| SAG | 0.3 µM | 0.5 µM | AML12 | (Yao et al., 2023) |
| TA | 40 µM | 5-100 µM | NIH 3T3 | (Wang et al., 2012) |
| RU-SKI 43 | 0.1 µM | 10 µM | NIH 3T3, COS-1, C3H10T1/2 | (Petrova et al., 2013) |
| Cyclo | 5 µM | 1-10 µM | Mouse hepatocytes, rat cholangiocytes | (Sato et al., 2018; Spormann et al., 2020) |
| Bud | 25 µM | 10-100 µM | NIH 3T3 | (Wang et al., 2012) |
| GANT61 | 0.5 µM | 2.5-40 µM | HT-29, HCT-116 | (Si et al., 2022) |
| Vismo | 0.1-10 µM | 10-40 µM (*in vitro*), 50 and 75 µM (*in vivo*) | human mast cells, human fibroblasts (*in vitro*) | (Biehs et al., 2018; Eibenschutz et al., 2021; Falduto et al., 2022; Tao et al., 2022) |

**Supplementary Table S2** Correlation coefficients and p-values of gene vs. protein correlation analysis. See Figure 1. (The data is available in the Excel file *Supplementary_Tables*)

**Supplementary Table S3** p-values of paired t-tests for qPCR analyses of Hh pathway-related genes with p-value < 0.05.

| Gene | Modulator | | | | | |
| --- | --- | --- | --- | --- | --- | --- |
|  | SAG | TA | RU-SKI 43 | Cyclo | Bud | GANT61 |
| *Ptch2* | *0.0982* | *0.9638* | 0.0075 | 0.0486 | 0.0337 | *0.4145* |
| *Fu* | 0.0489 | 0.0208 | *0.3447* | *0.2867* | *0.3474* | *0.6070* |

**Supplementary Table S4** Expression fold changes of Hh pathway-related genes and proteins detected in RNA-seq and proteomics and analyzed with IPA. N.d. means not detected. See Figure 2. (The data is available in the Excel file *Supplementary_Tables*)

**Supplementary Table S5** Expression fold changes of Wnt pathway-related genes and proteins detected in RNA-seq and proteomics and analyzed with IPA. N.d. means not detected. See Figure 2. (The data is available in the Excel file *Supplementary_Tables*)

**Supplementary Table S6** Log(2) Z-scores of canonical pathways regulated in RNA and protein datasets. See Figure 3. (The data is available in the Excel file *Supplementary_Tables*)

**Supplementary Table S7** Forward and reverse primer sequences used for qPCR of murine hepatocyte mRNA.

| **gene name** | **forward primer** | **reverse primer** |
| --- | --- | --- |
| *Apc* | AGGAGAATGCAGTCCTGTCC | TTGTGAGGTTCTGAAGTTGAGC |
| *Ctnnb1* | GCAGCAGCAGTCTTACTTGG | CCCTCATCTAGCGTCTCAGG |
| *Disp1* | CTTCAGCAGGAGGGGAGAC | TGGCGATGTAATTCCCCAGG |
| *Eif3f* | GACGTGTTTTAGCCCCAACC | ACAAGTTTCTCGTTGAGGGC |
| *Fu* | TGCCTCTCAGCCTTCTTAGG | TAAGAGCGCCCCATACCA |
| *Fzd6* | AAAGATCGGGACTGTGGGG | TGCAGTCTATCAGGCCAGTC |
| *Gli1* | Qiagen QuantiTect Primer Assay | |
| *Gli3* | Qiagen QuantiTect Primer Assay | |
| *Hhat* | CTGGGAGTCACTGTGGAGAG | TGAGCATGGAGGTAGAGCAG |
| *Hhip* | CTACTTGGGCCAGATGGAAG | CTCCAAGTAAGGCTCCTTGAAC |
| *Lgr4* | CATCCATTGTACACCTTCAACAG | GGCAGGCAGTGATGAACAAG |
| *Ppia* | CGAGCTCTGAGCACTGGAG | AGATGCCAGGACCTGTATGC |
| *Ptch1* | ACTCCAAAAGAAGAAGGCGC | CCAGAAGCAGTCCAAAGGTG |
| *Ptch2* | CCGAGTGGCTGTAATTGAGAC | CTGGAGGTGCAAGTCAAGTG |
| *Ror1* | TGTGCCGCAATAACCAGAAG | TCTTCCATGAAACGCACAGC |
| *Shh* | TCCAAAGCTCACATCCACTG | CTCCGGGACGTAAGTCCTTC |
| *Smo* | GCAAGCTCGTGCTCTGGT | GGGCATGTAGACAGCACACA |
| *Sufu* | CTTCCAGTCAGAGAACACCT | TTGGGCTGAATGTAACTCCT |
| *Wnt4* | CCGGGCACTCATGAATCTTC | GTGGCACCGTCAAACTTCTC |
| *Wnt5a* | CAAATAGGCAGCCGAGAGAC | GGAGTTCGTGGACGCTAGAG |

**Supplementary Table S8** Forward and reverse primer sequences used for qPCR of human hepatocyte mRNA.

| **gene name** | **forward primer** | **reverse primer** |
| --- | --- | --- |
| *ACTB* | GGATGATGATATCGCCGCG | AGGATGCCTCTCTTGCTCTG |
| *GLI1* | CCAGCCCCAACTCCACAGGC | GCAGCCATCCCAACGGCAGT |
| *GLI3* | GCTCCACGACCACTGAAAAG | TGTGGCTGCATAGTGATTGC |
| *HRPT1* | TGGCGTCGTGATTAGTGATG | GCTACAATGTGATGGCCTCC |
| *PPIA* | CAGCCTAAAGCCCAATATGC | GGGGTTTCATCATTTTGACC |
| *PTCH1* | AGAAGGTGCTAATGTCCTGAC | CCTGTTTCTGTGATAAGCTCTCC |
| *PTCH2* | GTACTTCTTTGCGGCGCTG | CTGTGGAGCTGGTGGACTC |
| *SMO* | GCTTCCGGGACTATGTGCTA | GCGATTCTTGATCTCACAGTCA |
| *SUFU* | AGATGGCTCCAACCTGAGTG | CAGGTTTGCTGTTGATCTCG |

# Supplementary Table S9 Binding sites of GLI1 in hepatocytes of male C57BL/6N mice identified by ChIP-seq associated with metabolic pathways, N = 4. Genes highlighted in yellow were published in Ott et al. 2022 (Ott et al., 2022) (The data is available in the Excel file *Supplementary_Tables*)

# References

Azimifar, S. B., Nagaraj, N., Cox, J., et al. (2014). Cell-type-resolved quantitative proteomics of murine liver. *Cell Metab* 20, 1076–1087. doi: 10.1016/j.cmet.2014.11.002

Biehs, B., Dijkgraaf, G. J. P., Piskol, R., et al. (2018). A cell identity switch allows residual BCC to survive Hedgehog pathway inhibition. *Nature* 562, 429–433. doi: 10.1038/s41586-018-0596-y

Deutsch, E. W., Bandeira, N., Perez-Riverol, Y., et al. (2023). The ProteomeXchange consortium at 10 years: 2023 update. *Nucleic Acids Res* 51, D1539-D1548. doi: 10.1093/nar/gkac1040

Eibenschutz, L., Caputo, S., Camera, E., et al. (2021). Evaluation of Hedgehog Pathway Inhibition on Nevoid Basal Cell Carcinoma Syndrome Fibroblasts and Basal Cell Carcinoma-Associated Fibroblasts: Are Vismodegib and Sonidegib Useful to Target Cancer-Prone Fibroblasts? *Cancers (Basel)* 13. doi: 10.3390/cancers13225858

Falduto, G. H., Pfeiffer, A., Zhang, Q., et al. (2022). A Critical Function for the Transcription Factors GLI1 and GLI2 in the Proliferation and Survival of Human Mast Cells. *Front Immunol* 13, 841045. doi: 10.3389/fimmu.2022.841045

Loroch, S., Kopczynski, D., Schneider, A. C., et al. (2022). Toward Zero Variance in Proteomics Sample Preparation: Positive-Pressure FASP in 96-Well Format (PF96) Enables Highly Reproducible, Time- and Cost-Efficient Analysis of Sample Cohorts. *J Proteome Res* 21, 1181–1188. doi: 10.1021/acs.jproteome.1c00706

Ott, F., Körner, C., Werner, K., et al. (2022). Hepatic Hedgehog Signaling Participates in the Crosstalk between Liver and Adipose Tissue in Mice by Regulating FGF21. *Cells* 11. doi: 10.3390/cells11101680

Petrova, E., Rios-Esteves, J., Ouerfelli, O., et al. (2013). Inhibitors of Hedgehog acyltransferase block Sonic Hedgehog signaling. *Nat Chem Biol* 9, 247–249. doi: 10.1038/nchembio.1184

Petrova, R., and Joyner, A. L. (2014). Roles for Hedgehog signaling in adult organ homeostasis and repair. *Development* 141, 3445–3457. doi: 10.1242/dev.083691

Sato, Y., Yamamura, M., Sasaki, M., et al. (2018). Blockade of Hedgehog Signaling Attenuates Biliary Cystogenesis in the Polycystic Kidney (PCK) Rat. *Am J Pathol* 188, 2251–2263. doi: 10.1016/j.ajpath.2018.06.014

Si, Y., Li, L., Zhang, W., et al. (2022). GANT61 exerts anticancer cell and anticancer stem cell capacity in colorectal cancer by blocking the Wnt/β‑catenin and Notch signalling pathways. *Oncol Rep* 48. doi: 10.3892/or.2022.8397

Spormann, L., Rennert, C., Kolbe, E., et al. (2020). Cyclopamine and Rapamycin Synergistically Inhibit mTOR Signalling in Mouse Hepatocytes, Revealing an Interaction of Hedgehog and mTor Signalling in the Liver. *Cells* 9. doi: 10.3390/cells9081817

Tao, J., Chen, Y., Zhuang, Y., et al. (2022). Inhibition of Hedgehog Delays Liver Regeneration through Disrupting the Cell Cycle. *Curr Issues Mol Biol* 44, 470–482. doi: 10.3390/cimb44020032

Teperino, R., Aberger, F., Esterbauer, H., et al. (2014). Canonical and non-canonical Hedgehog signalling and the control of metabolism. *Semin Cell Dev Biol* 0, 81–92. doi: 10.1016/j.semcdb.2014.05.007

Wang, Y., Davidow, L., Arvanites, A. C., et al. (2012). Glucocorticoid compounds modify smoothened localization and hedgehog pathway activity. *Chem Biol* 19, 972–982. doi: 10.1016/j.chembiol.2012.06.012

Wu, F., Zhang, Y., Sun, B., et al. (2017). Hedgehog Signaling: From Basic Biology to Cancer Therapy. *Cell Chem Biol* 24, 252–280. doi: 10.1016/j.chembiol.2017.02.010

Yao, Y., Li, T., Yu, T., et al. (2023). Hedgehog signal activates AMPK via Smoothened to promote autophagy and lipid degradation in hepatocytes. *Biochem Cell Biol*. doi: 10.1139/bcb-2022-0345
