# Supplementary material for: Impact of Hedgehog modulators on signaling pathways in primary murine and human hepatocytes in vitro: insights into liver metabolism
Source: Arch Toxicol. 2024 Dec 23;99(3):1105–16. doi: 10.1007/s00204-024-03931-y (PMC11821798; doi:10.1007/s00204-024-03931-y)

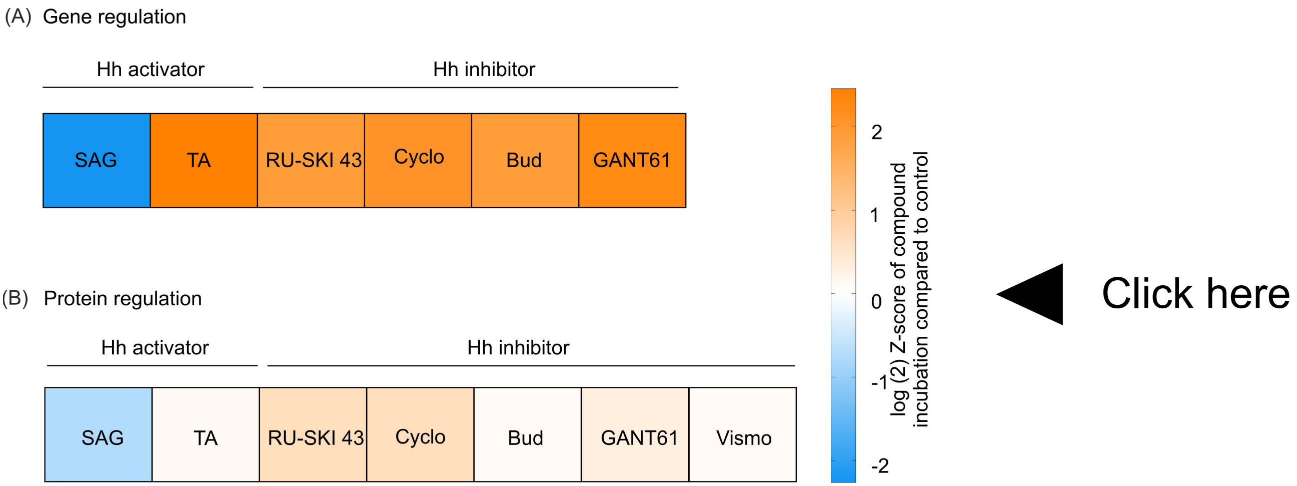

Supplement Figure 4 - interactive pdf:

Hh signaling pathway and its detailed dynamics during culture of primary hepatocytes with Hh modulators. The heatmap shows the activation Z-score analysis based on RNA-Seq. (top) and proteomics (down) of male hepatocytes incubated with the Hh modulators compared to the control, respectively. The activation Z-score was calculated with IPA software. The p-value cutoff of 0.05 was used for calculation. A click on the colored squares reveals the detailed pathway analysis of the Hh pathway of compound incubation compared to control incubation after 48 h done by IPA.

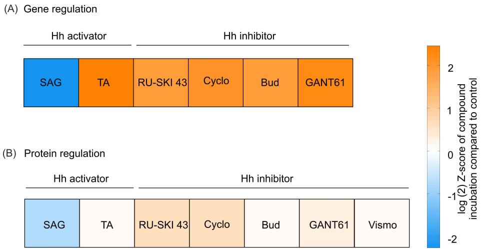

SAG vs control - gene expression

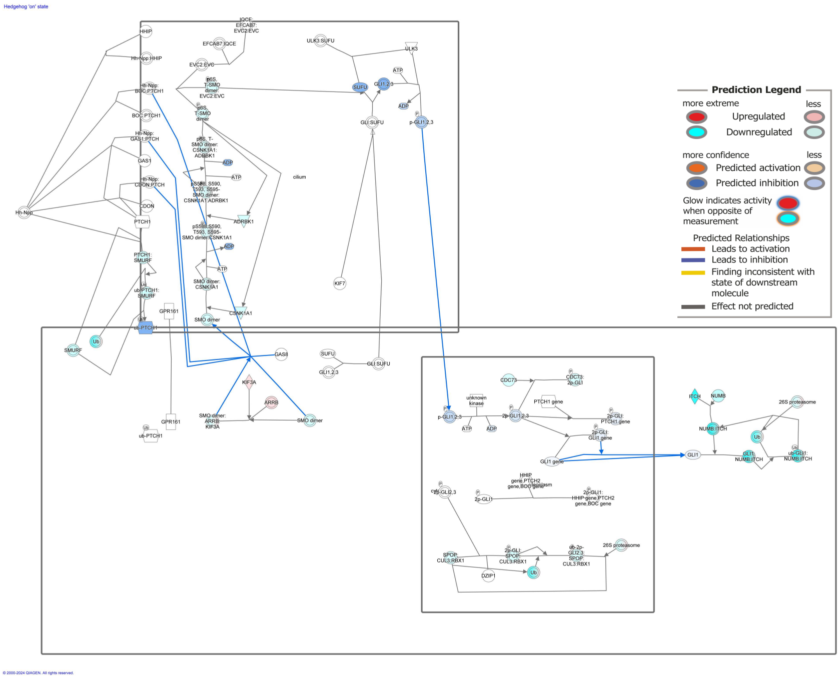

Supplement Figure 4 - interactive pdf:

Hh signaling pathway and its detailed dynamics during culture of primary hepatocytes with Hh modulators. The heatmap shows the activation Z-score analysis based on RNA-Seq. (top) and proteomics (down) of male hepatocytes incubated with the Hh modulators compared to the control, respectively. The activation Z-score was calculated with IPA software. The p-value cutoff of 0.05 was used for calculation. A click on the colored squares reveals the detailed pathway analysis of the Hh pathway of compound incubation compared to control incubation after 48 h done by IPA. Nodes with color gradients represent complexes whose individual components are regulated differently. Lines symbolize direct interaction. Dashed lines symbolize indirect interaction.

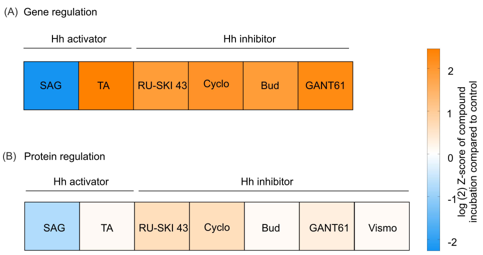

TA vs control - gene expression

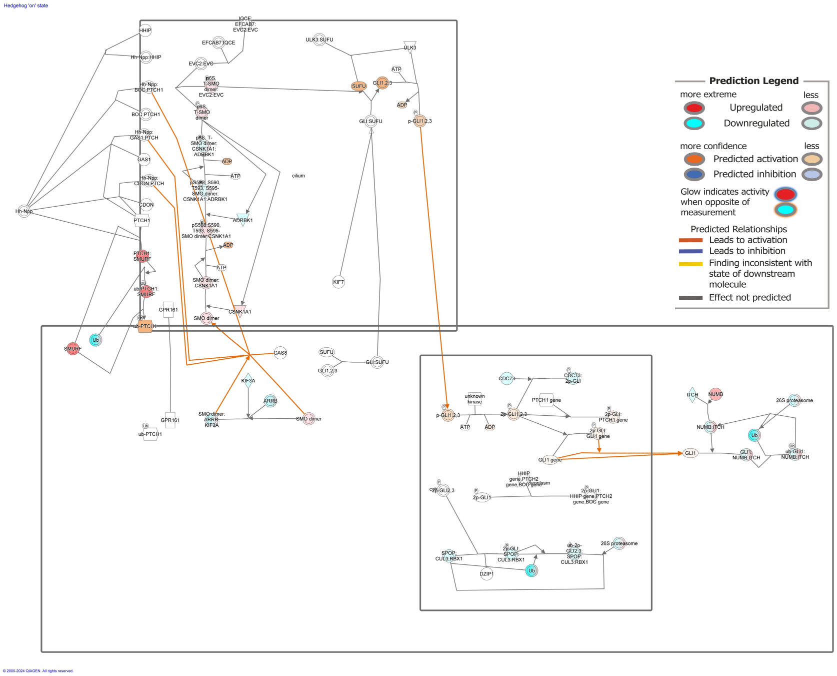

Supplement Figure 4 - interactive pdf:

Hh signaling pathway and its detailed dynamics during culture of primary hepatocytes with Hh modulators. The heatmap shows the activation Z-score analysis based on RNA-Seq. (top) and proteomics (down) of male hepatocytes incubated with the Hh modulators compared to the control, respectively. The activation Z-score was calculated with IPA software. The p-value cutoff of 0.05 was used for calculation. A click on the colored squares reveals the detailed pathway analysis of the Hh pathway of compound incubation compared to control incubation after 48 h done by IPA. Nodes with color gradients represent complexes whose individual components are regulated differently. Lines symbolize direct interaction. Dashed lines symbolize indirect interaction.

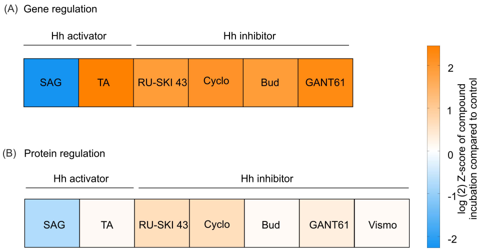

Supplement Figure 4 - interactive pdf:

Hh signaling pathway and its detailed dynamics during culture of primary hepatocytes with Hh modulators. The heatmap shows the activation Z-score analysis based on RNA-Seq. (top) and proteomics (down) of male hepatocytes incubated with the Hh modulators compared to the control, respectively. The activation Z-score was calculated with IPA software. The p-value cutoff of 0.05 was used for calculation. A click on the colored squares reveals the detailed pathway analysis of the Hh pathway of compound incubation compared to control incubation after 48 h done by IPA. Nodes with color gradients represent complexes whose individual components are regulated differently. Lines symbolize direct interaction. Dashed lines symbolize indirect interaction.

RU-SKI 43 vs control - gene expression

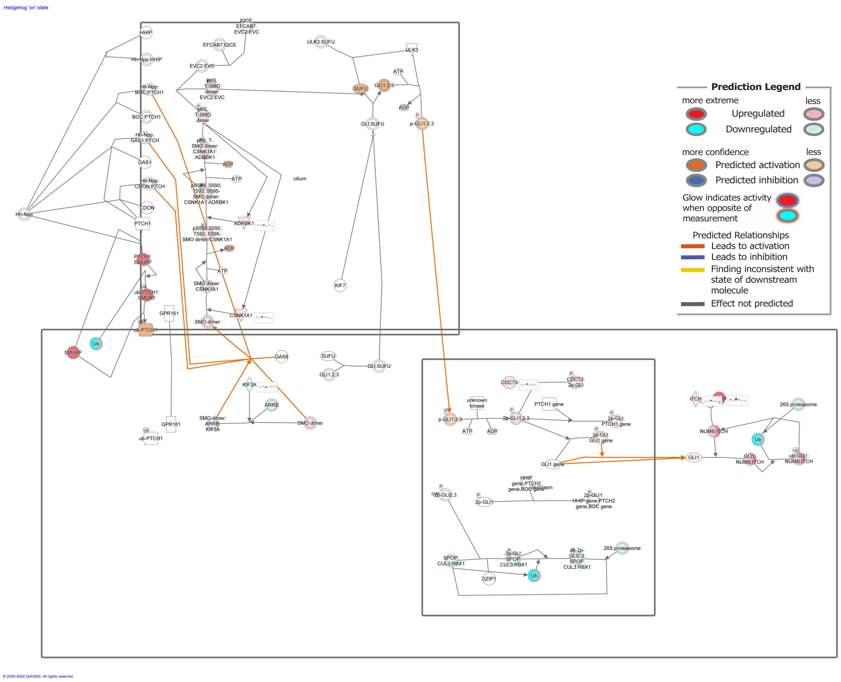

Cyclo vs control - gene expression

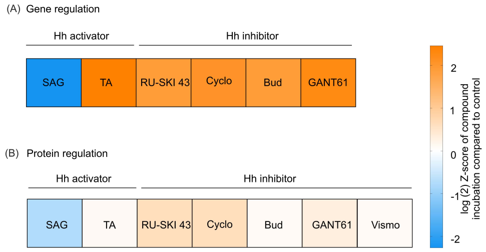

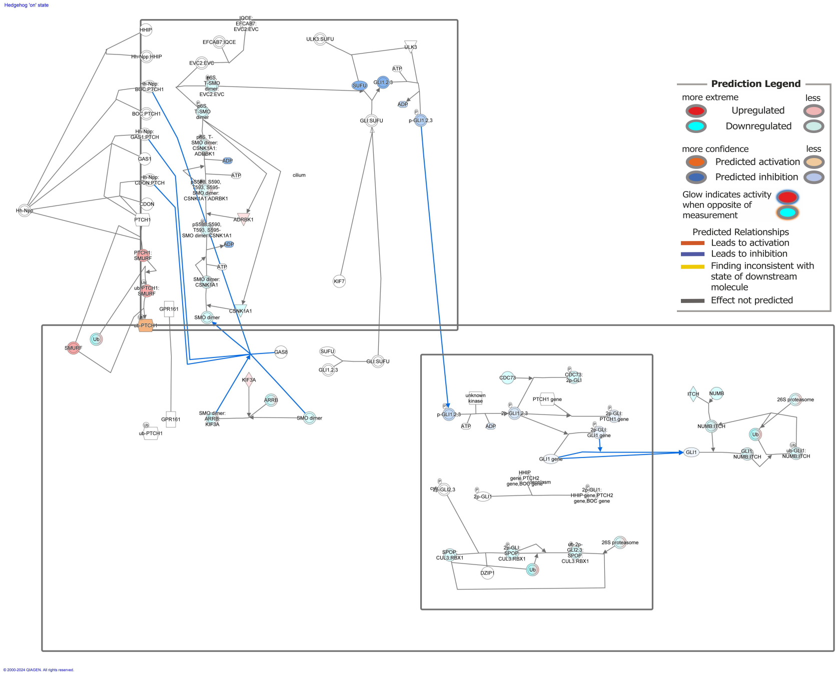

Supplement Figure 4 - interactive pdf:

Hh signaling pathway and its detailed dynamics during culture of primary hepatocytes with Hh modulators. The heatmap shows the activation Z-score analysis based on RNA-Seq. (top) and proteomics (down) of male hepatocytes incubated with the Hh modulators compared to the control, respectively. The activation Z-score was calculated with IPA software. The p-value cutoff of 0.05 was used for calculation. A click on the colored squares reveals the detailed pathway analysis of the Hh pathway of compound incubation compared to control incubation after 48 h done by IPA. Nodes with color gradients represent complexes whose individual components are regulated differently. Lines symbolize direct interaction. Dashed lines symbolize indirect interaction.

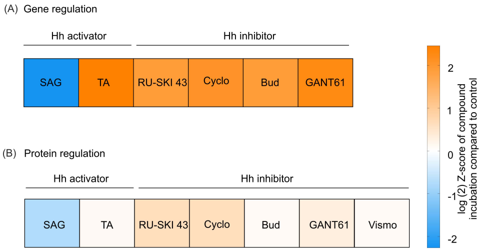

Supplement Figure 4 - interactive pdf:

Hh signaling pathway and its detailed dynamics during culture of primary hepatocytes with Hh modulators. The heatmap shows the activation Z-score analysis based on RNA-Seq. (top) and proteomics (down) of male hepatocytes incubated with the Hh modulators compared to the control, respectively. The activation Z-score was calculated with IPA software. The p-value cutoff of 0.05 was used for calculation. A click on the colored squares reveals the detailed pathway analysis of the Hh pathway of compound incubation compared to control incubation after 48 h done by IPA. Nodes with color gradients represent complexes whose individual components are regulated differently. Lines symbolize direct interaction. Dashed lines symbolize indirect interaction.

Bud vs control - gene expression

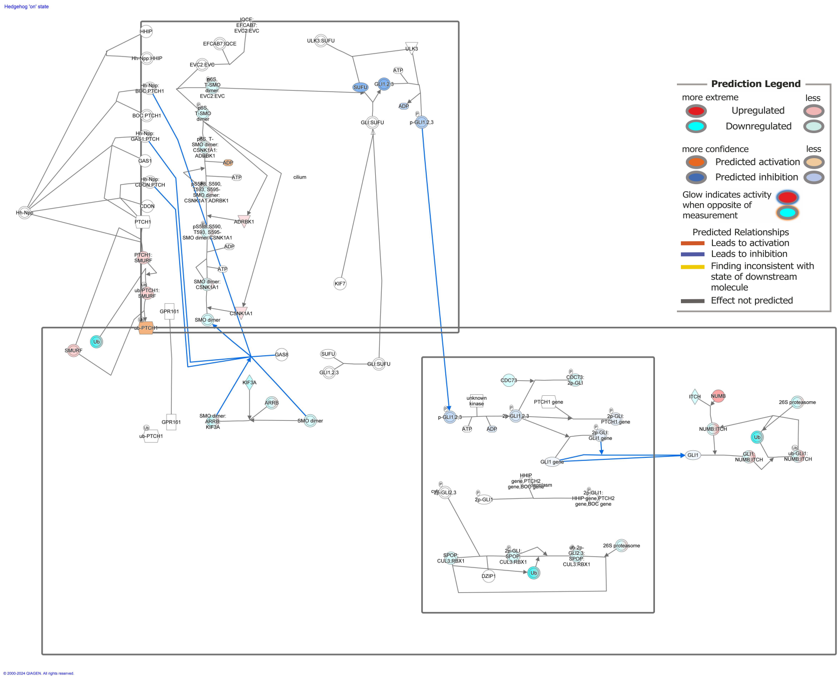

GANT61 vs control - gene expression

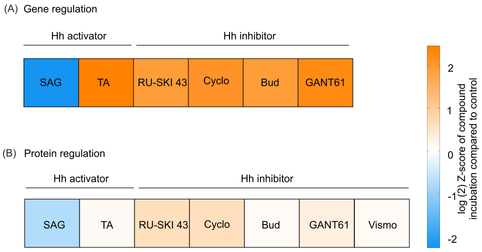

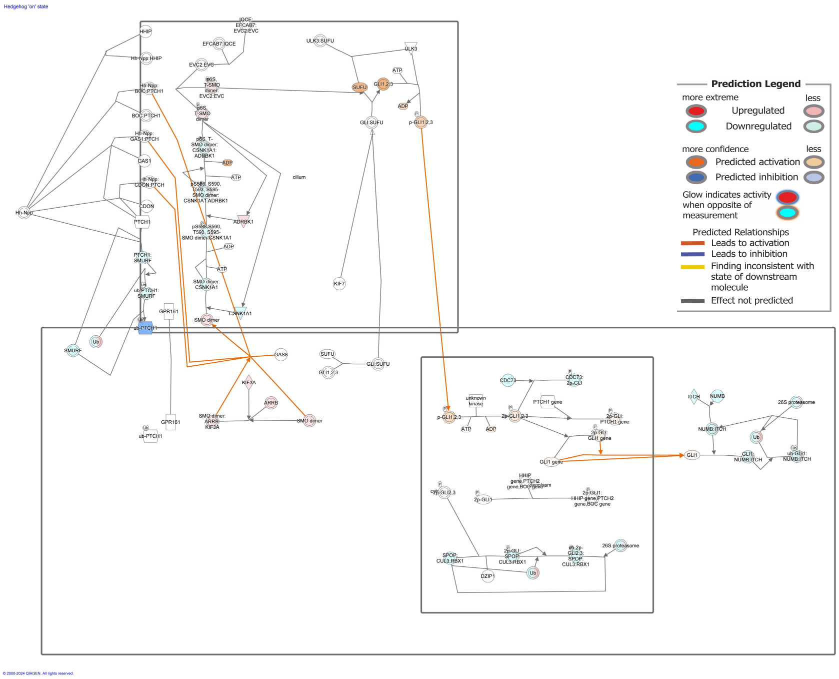

Supplement Figure 4 - interactive pdf:

Hh signaling pathway and its detailed dynamics during culture of primary hepatocytes with Hh modulators. The heatmap shows the activation Z-score analysis based on RNA-Seq. (top) and proteomics (down) of male hepatocytes incubated with the Hh modulators compared to the control, respectively. The activation Z-score was calculated with IPA software. The p-value cutoff of 0.05 was used for calculation. A click on the colored squares reveals the detailed pathway analysis of the Hh pathway of compound incubation compared to control incubation after 48 h done by IPA. Nodes with color gradients represent complexes whose individual components are regulated differently. Lines symbolize direct interaction. Dashed lines symbolize indirect interaction.

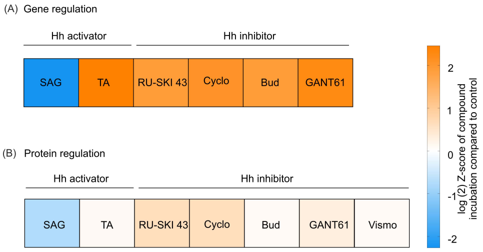

Supplement Figure 4 - interactive pdf:

Hh signaling pathway and its detailed dynamics during culture of primary hepatocytes with Hh modulators. The heatmap shows the activation Z-score analysis based on RNA-Seq. (top) and proteomics (down) of male hepatocytes incubated with the Hh modulators compared to the control, respectively. The activation Z-score was calculated with IPA software. The p-value cutoff of 0.05 was used for calculation. A click on the colored squares reveals the detailed pathway analysis of the Hh pathway of compound incubation compared to control incubation after 48 h done by IPA. Nodes with color gradients represent complexes whose individual components are regulated differently. Lines symbolize direct interaction. Dashed lines symbolize indirect interaction.

SAG vs control - protein translation

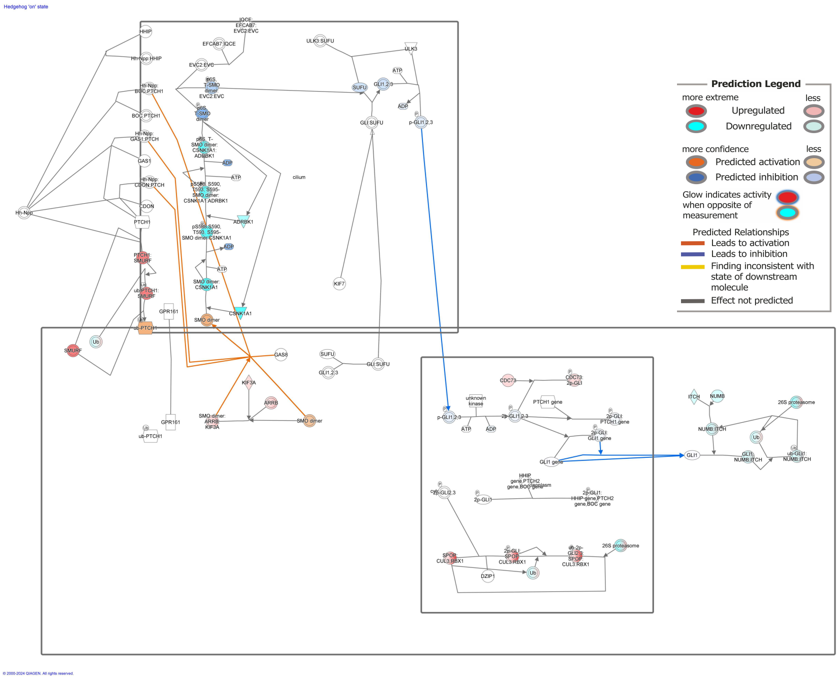

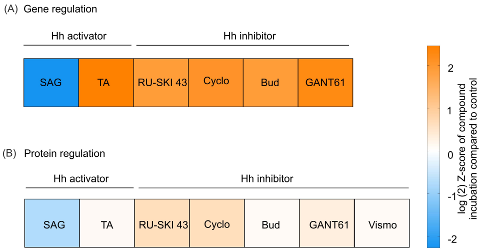

TA vs control - protein translation

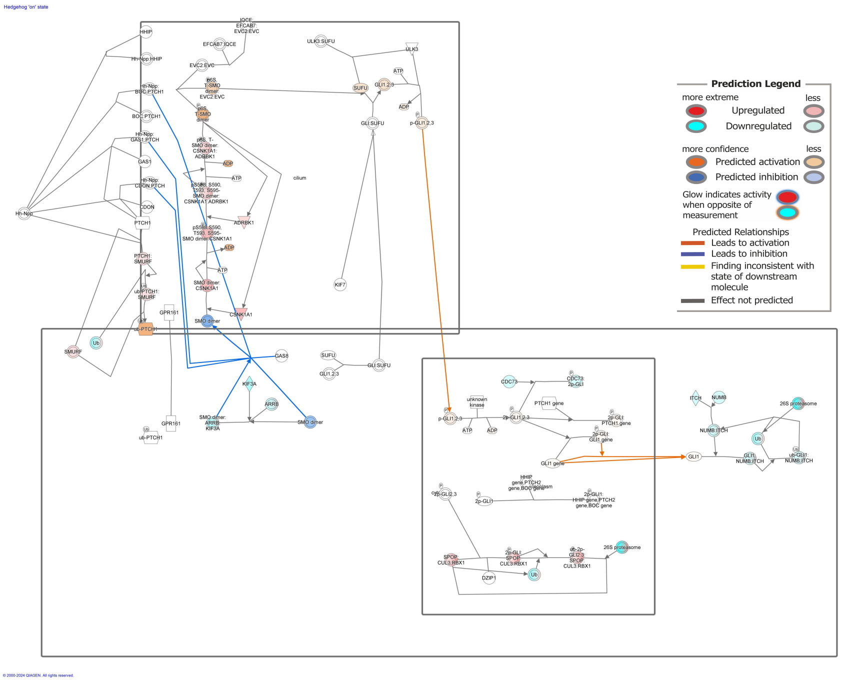

Supplement Figure 4 - interactive pdf:

Hh signaling pathway and its detailed dynamics during culture of primary hepatocytes with Hh modulators. The heatmap shows the activation Z-score analysis based on RNA-Seq. (top) and proteomics (down) of male hepatocytes incubated with the Hh modulators compared to the control, respectively. The activation Z-score was calculated with IPA software. The p-value cutoff of 0.05 was used for calculation. A click on the colored squares reveals the detailed pathway analysis of the Hh pathway of compound incubation compared to control incubation after 48 h done by IPA. Nodes with color gradients represent complexes whose individual components are regulated differently. Lines symbolize direct interaction. Dashed lines symbolize indirect interaction.

RU-SKI 43 vs control - protein translation

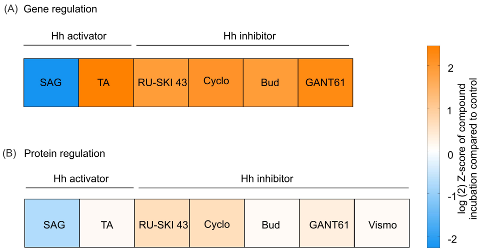

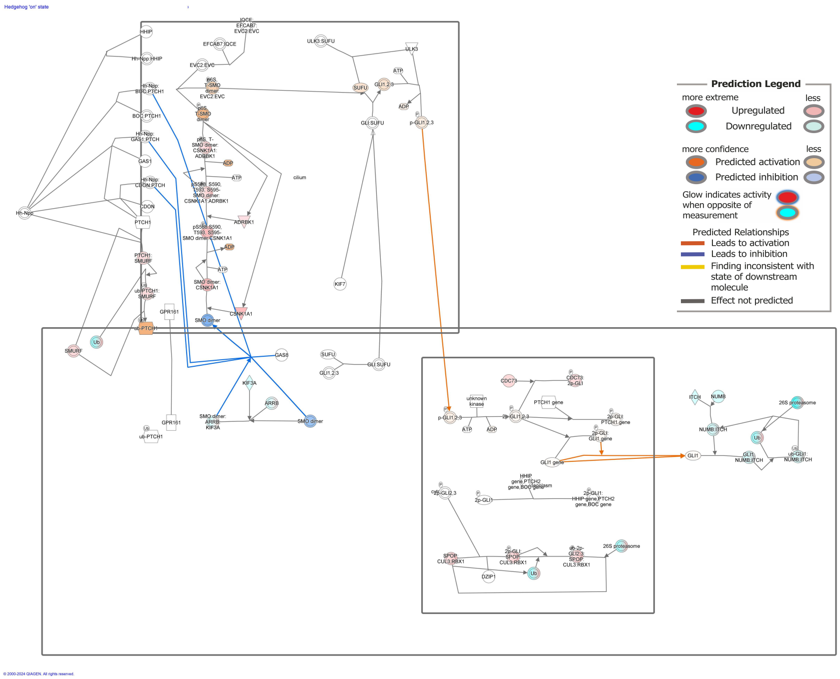

Supplement Figure 4 - interactive pdf:

Hh signaling pathway and its detailed dynamics during culture of primary hepatocytes with Hh modulators. The heatmap shows the activation Z-score analysis based on RNA-Seq. (top) and proteomics (down) of male hepatocytes incubated with the Hh modulators compared to the control, respectively. The activation Z-score was calculated with IPA software. The p-value cutoff of 0.05 was used for calculation. A click on the colored squares reveals the detailed pathway analysis of the Hh pathway of compound incubation compared to control incubation after 48 h done by IPA. Nodes with color gradients represent complexes whose individual components are regulated differently. Lines symbolize direct interaction. Dashed lines symbolize indirect interaction.

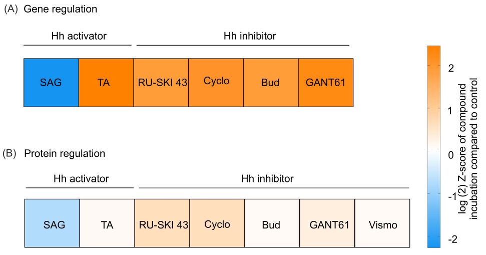

Cyclo vs control - protein translation

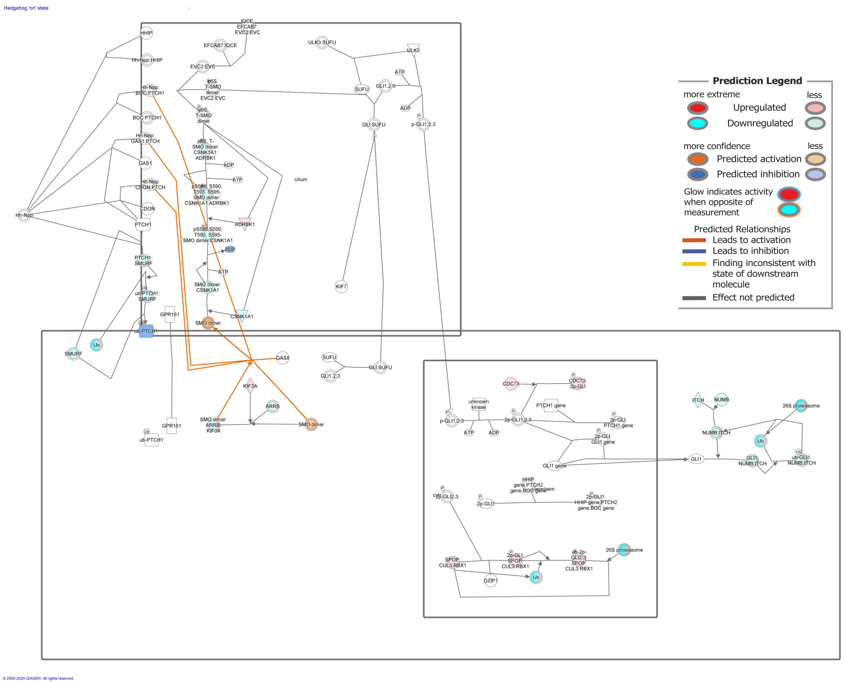

Supplement Figure 4 - interactive pdf:

Hh signaling pathway and its detailed dynamics during culture of primary hepatocytes with Hh modulators. The heatmap shows the activation Z-score analysis based on RNA-Seq. (top) and proteomics (down) of male hepatocytes incubated with the Hh modulators compared to the control, respectively. The activation Z-score was calculated with IPA software. The p-value cutoff of 0.05 was used for calculation. A click on the colored squares reveals the detailed pathway analysis of the Hh pathway of compound incubation compared to control incubation after 48 h done by IPA. Nodes with color gradients represent complexes whose individual components are regulated differently. Lines symbolize direct interaction. Dashed lines symbolize indirect interaction.

Bud vs control - protein translation

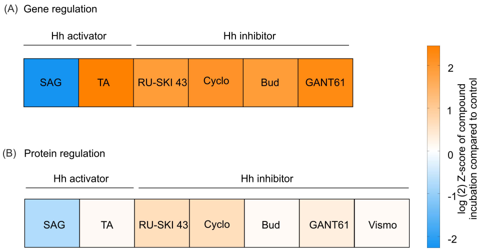

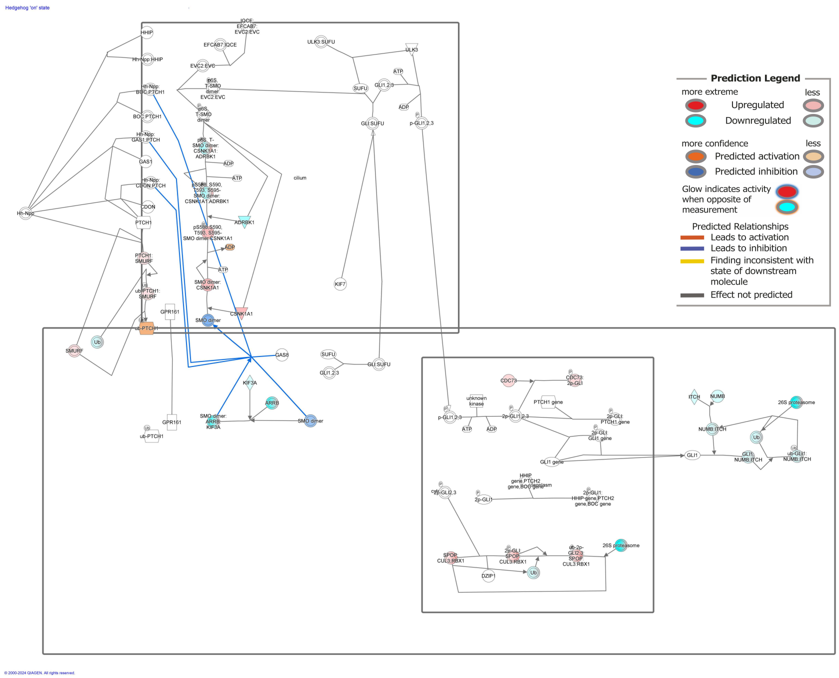

Supplement Figure 4 - interactive pdf:

Hh signaling pathway and its detailed dynamics during culture of primary hepatocytes with Hh modulators. The heatmap shows the activation Z-score analysis based on RNA-Seq. (top) and proteomics (down) of male hepatocytes incubated with the Hh modulators compared to the control, respectively. The activation Z-score was calculated with IPA software. The p-value cutoff of 0.05 was used for calculation. A click on the colored squares reveals the detailed pathway analysis of the Hh pathway of compound incubation compared to control incubation after 48 h done by IPA. Nodes with color gradients represent complexes whose individual components are regulated differently. Lines symbolize direct interaction. Dashed lines symbolize indirect interaction.

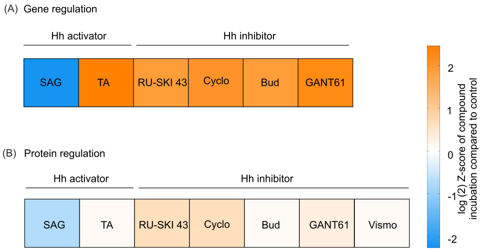

GANT61 vs control - protein translation

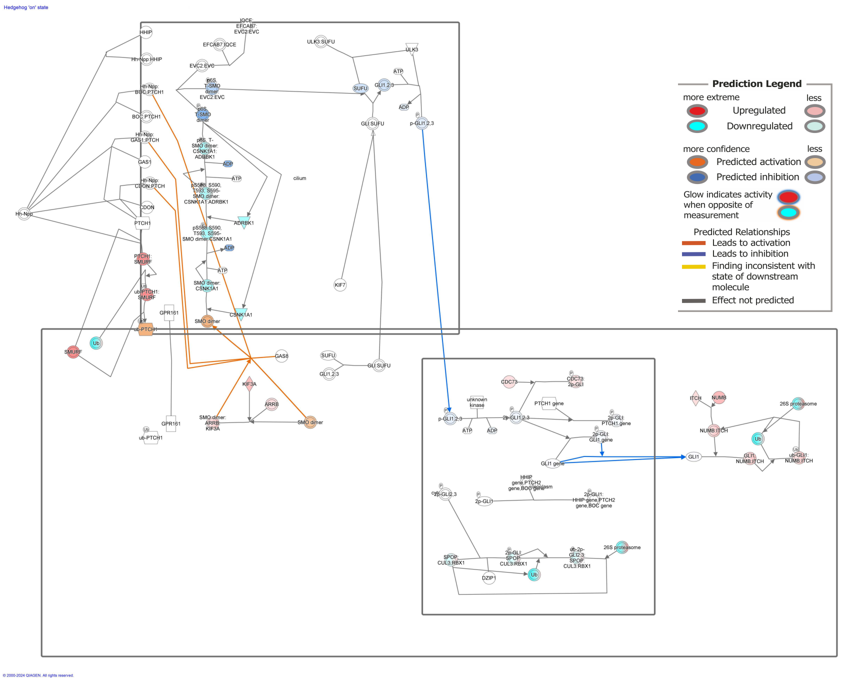

Supplement Figure 4 - interactive pdf:

Hh signaling pathway and its detailed dynamics during culture of primary hepatocytes with Hh modulators. The heatmap shows the activation Z-score analysis based on RNA-Seq. (top) and proteomics (down) of male hepatocytes incubated with the Hh modulators compared to the control, respectively. The activation Z-score was calculated with IPA software. The p-value cutoff of 0.05 was used for calculation. A click on the colored squares reveals the detailed pathway analysis of the Hh pathway of compound incubation compared to control incubation after 48 h done by IPA. Nodes with color gradients represent complexes whose individual components are regulated differently. Lines symbolize direct interaction. Dashed lines symbolize indirect interaction.

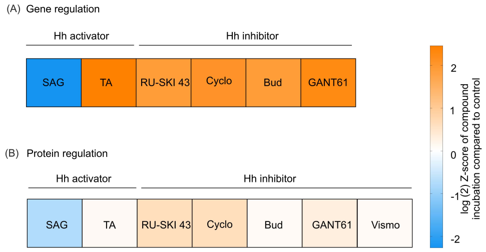

Supplement Figure 4 - interactive pdf:

Hh signaling pathway and its detailed dynamics during culture of primary hepatocytes with Hh modulators. The heatmap shows the activation Z-score analysis based on RNA-Seq. (top) and proteomics (down) of male hepatocytes incubated with the Hh modulators compared to the control, respectively. The activation Z-score was calculated with IPA software. The p-value cutoff of 0.05 was used for calculation. A click on the colored squares reveals the detailed pathway analysis of the Hh pathway of compound incubation compared to control incubation after 48 h done by IPA. Nodes with color gradients represent complexes whose individual components are regulated differently. Lines symbolize direct interaction. Dashed lines symbolize indirect interaction.

Vismo vs control - protein translation

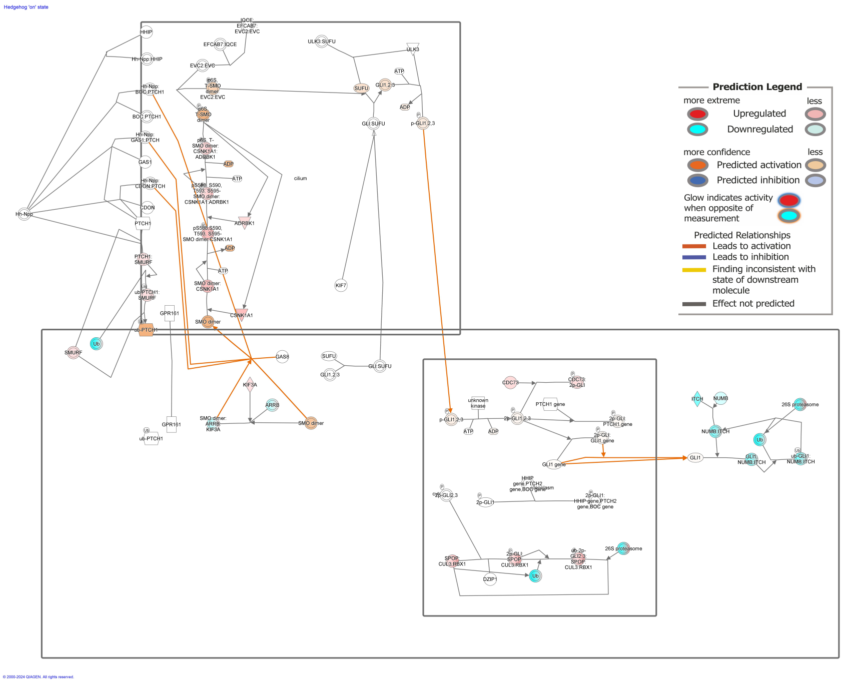

Supplement: Supplementary file 2 — Supplementary file2 (PDF 7580 KB) [file 204_2024_3931_MOESM2_ESM.pdf]
