# Supplementary material for: Impact of Hedgehog modulators on signaling pathways in primary murine and human hepatocytes in vitro: insights into liver metabolism
Source: Arch Toxicol. 2024 Dec 23;99(3):1105–16. doi: 10.1007/s00204-024-03931-y (PMC11821798; doi:10.1007/s00204-024-03931-y)
Supplement: Supplementary file 3 — Supplementary file3 (PDF 9949 KB) [file 204_2024_3931_MOESM3_ESM.pdf]

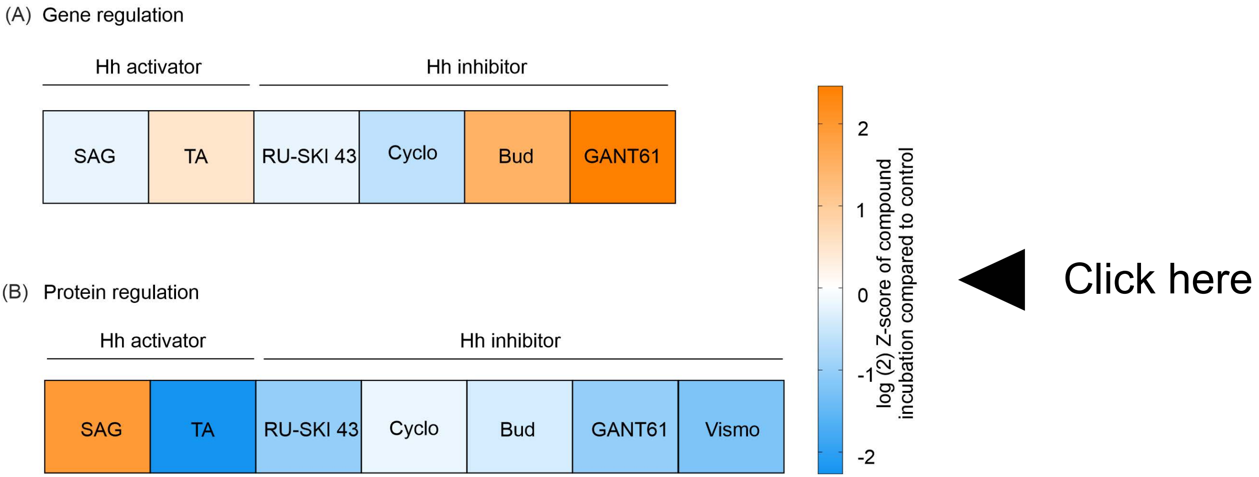

Supplement Figure 5 - interactive pdf:

Wnt signaling pathway and its detailed dynamics during culture of primary hepatocytes with Hh modulators. The heatmap shows the activation Z-score analysis based on RNA-Seq.
(top) and proteomics (down) of male hepatocytes incubated with the Hh modulators compared to the control, respectively. The activation Z-score was calculated with IPA software. The p-value cutoff of 0.05 was used for calculation. A click on the colored squares reveals the detailed pathway analysis of the Wnt pathway of compound incubation compared to control incubation after 48 h done by IPA.

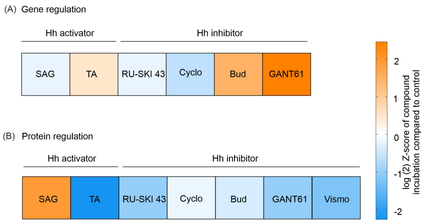

SAG vs control - gene expression

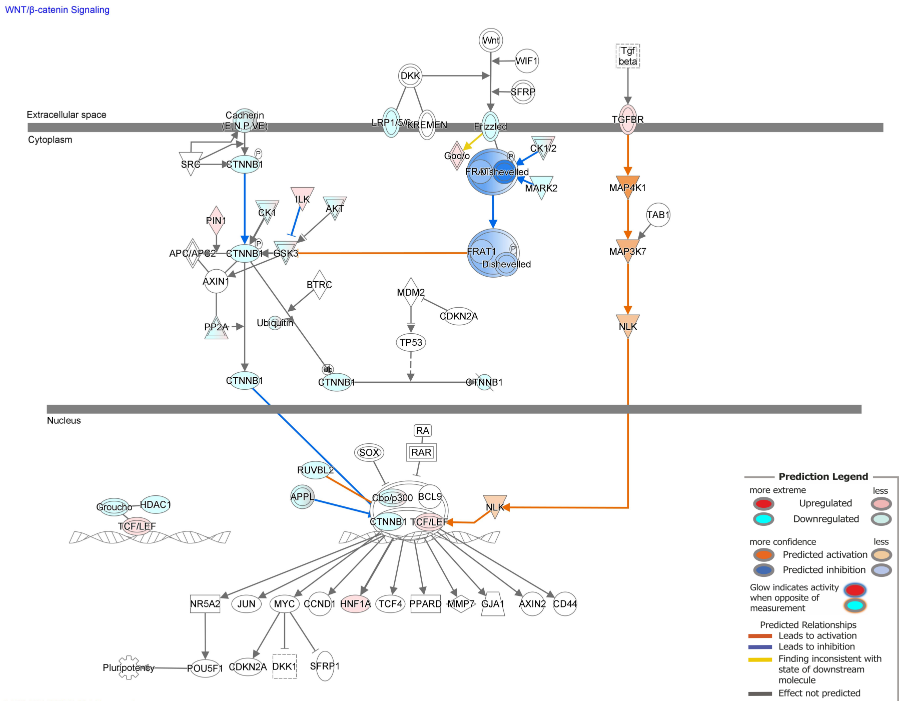

© 2000-2024 QIAGEN. All rights reserved.

Supplement Figure 5 - interactive pdf:

Wnt signaling pathway and its detailed dynamics during culture of primary hepatocytes with Hh modulators. The heatmap shows the activation Z-score analysis based on RNA-Seq. (top) and proteomics (down) of male hepatocytes incubated with the Hh modulators compared to the control, respectively. The activation Z-score was calculated with IPA software. The p-value cutoff of 0.05 was used for calculation. A click on the colored squares reveals the detailed pathway analysis of the Wnt pathway of compound incubation compared to control incubation after 48 h done by IPA. Nodes with color gradients represent complexes whose individual components are regulated differently. Lines symbolize direct interaction. Dashed lines symbolize indirect interaction.

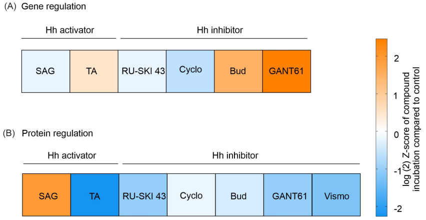

TA vs control - gene expression

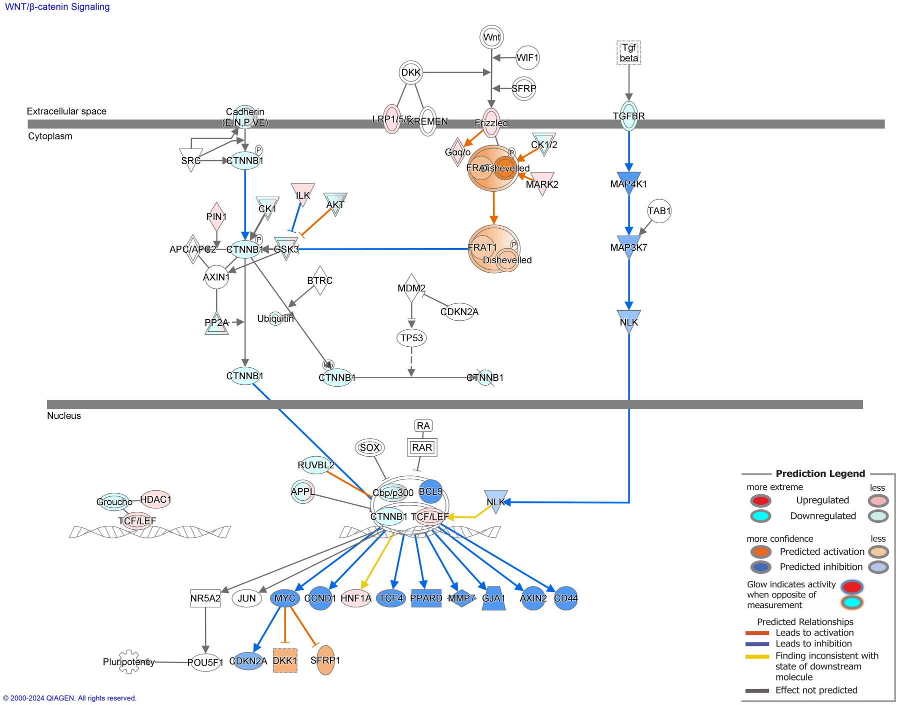

Supplement Figure 5 - interactive pdf:

Wnt signaling pathway and its detailed dynamics during culture of primary hepatocytes with Hh modulators. The heatmap shows the activation Z-score analysis based on RNA-Seq. (top) and proteomics (down) of male hepatocytes incubated with the Hh modulators compared to the control, respectively. The activation Z-score was calculated with IPA software. The p-value cutoff of 0.05 was used for calculation. A click on the colored squares reveals the detailed pathway analysis of the Wnt pathway of compound incubation compared to control incubation after 48 h done by IPA. Nodes with color gradients represent complexes whose individual components are regulated differently. Lines symbolize direct interaction. Dashed lines symbolize indirect interaction.

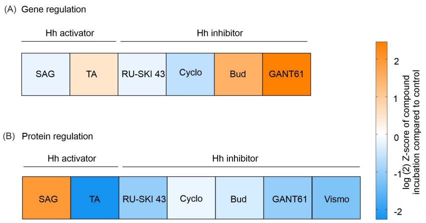

RU-SKI 43 vs control - gene expression

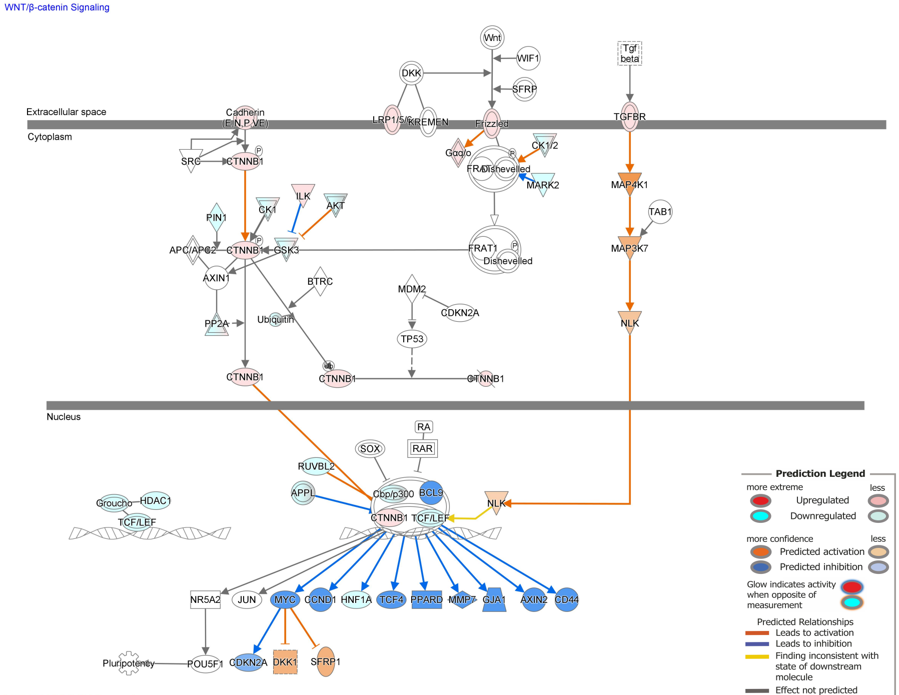

© 2000-2024 QIAGEN. All rights reserved.

Supplement Figure 5 - interactive pdf:

Wnt signaling pathway and its detailed dynamics during culture of primary hepatocytes with Hh modulators. The heatmap shows the activation Z-score analysis based on RNA-Seq. (top) and proteomics (down) of male hepatocytes incubated with the Hh modulators compared to the control, respectively. The activation Z-score was calculated with IPA software. The p-value cutoff of 0.05 was used for calculation. A click on the colored squares reveals the detailed pathway analysis of the Wnt pathway of compound incubation compared to control incubation after 48 h done by IPA. Nodes with color gradients represent complexes whose individual components are regulated differently. Lines symbolize direct interaction. Dashed lines symbolize indirect interaction.

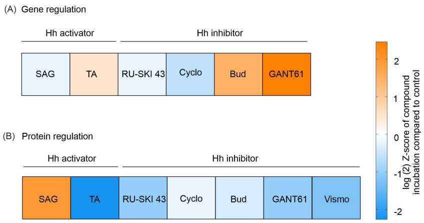

Cyclo vs control - gene expression

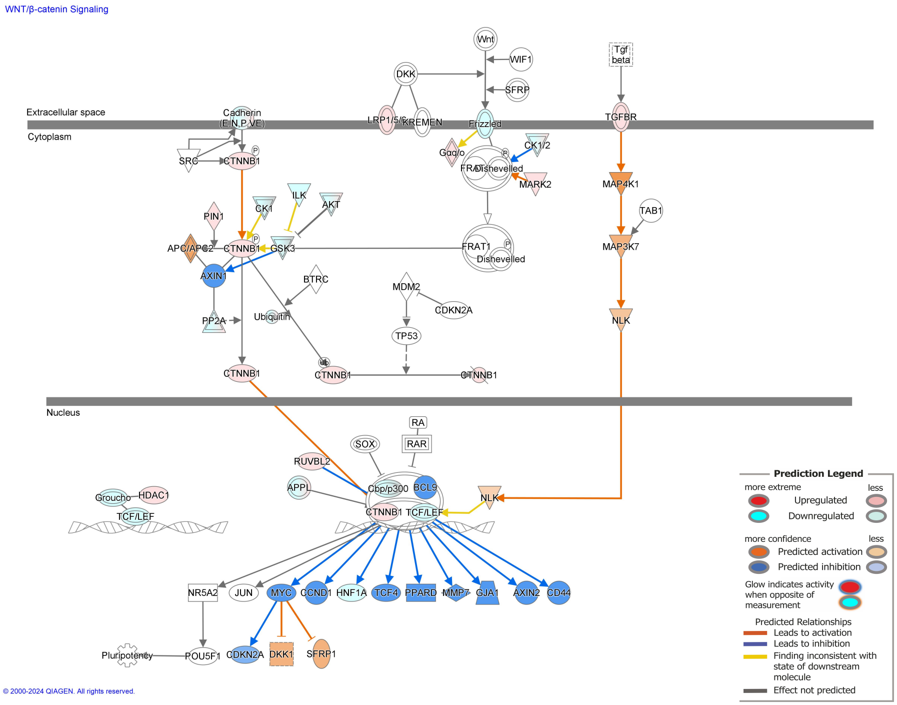

© 2000-2024 QIAGEN. All rights reserved.

Supplement Figure 5 - interactive pdf:

Wnt signaling pathway and its detailed dynamics during culture of primary hepatocytes with Hh modulators. The heatmap shows the activation Z-score analysis based on RNA-Seq. (top) and proteomics (down) of male hepatocytes incubated with the Hh modulators compared to the control, respectively. The activation Z-score was calculated with IPA software. The p-value cutoff of 0.05 was used for calculation. A click on the colored squares reveals the detailed pathway analysis of the Wnt pathway of compound incubation compared to control incubation after 48 h done by IPA. Nodes with color gradients represent complexes whose individual components are regulated differently. Lines symbolize direct interaction. Dashed lines symbolize indirect interaction.

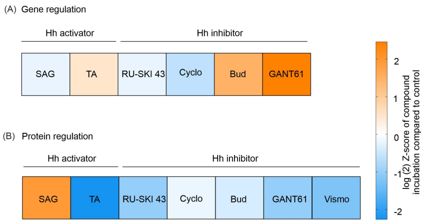

Bud vs control - gene expression

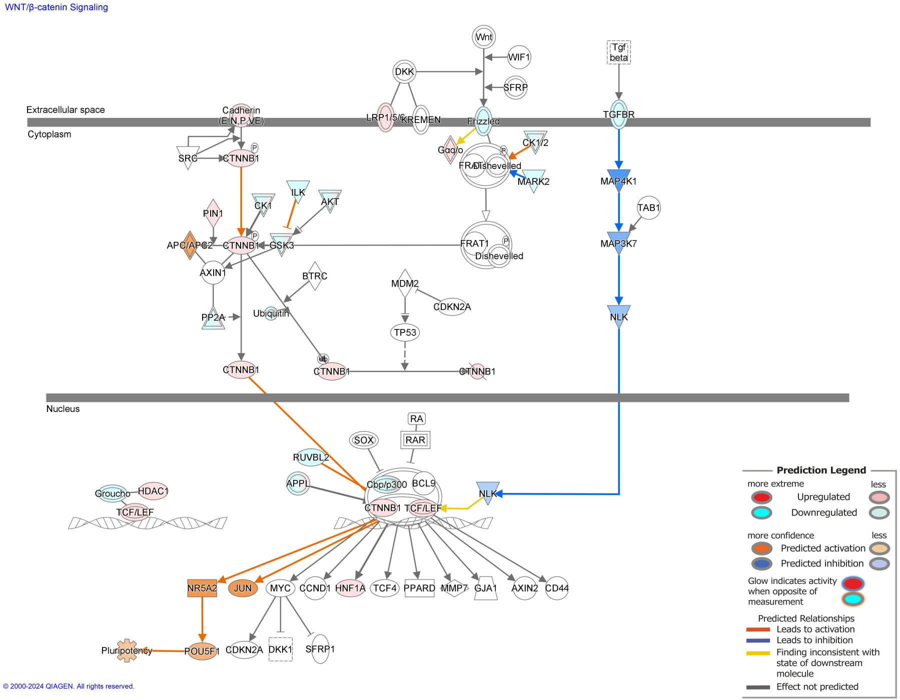

© 2000-2024 QIAGEN. All rights reserved.

Supplement Figure 5 - interactive pdf:

Wnt signaling pathway and its detailed dynamics during culture of primary hepatocytes with Hh modulators. The heatmap shows the activation Z-score analysis based on RNA-Seq. (top) and proteomics (down) of male hepatocytes incubated with the Hh modulators compared to the control, respectively. The activation Z-score was calculated with IPA software. The p-value cutoff of 0.05 was used for calculation. A click on the colored squares reveals the detailed pathway analysis of the Wnt pathway of compound incubation compared to control incubation after 48 h done by IPA. Nodes with color gradients represent complexes whose individual components are regulated differently. Lines symbolize direct interaction. Dashed lines symbolize indirect interaction.

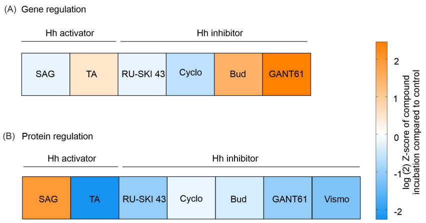

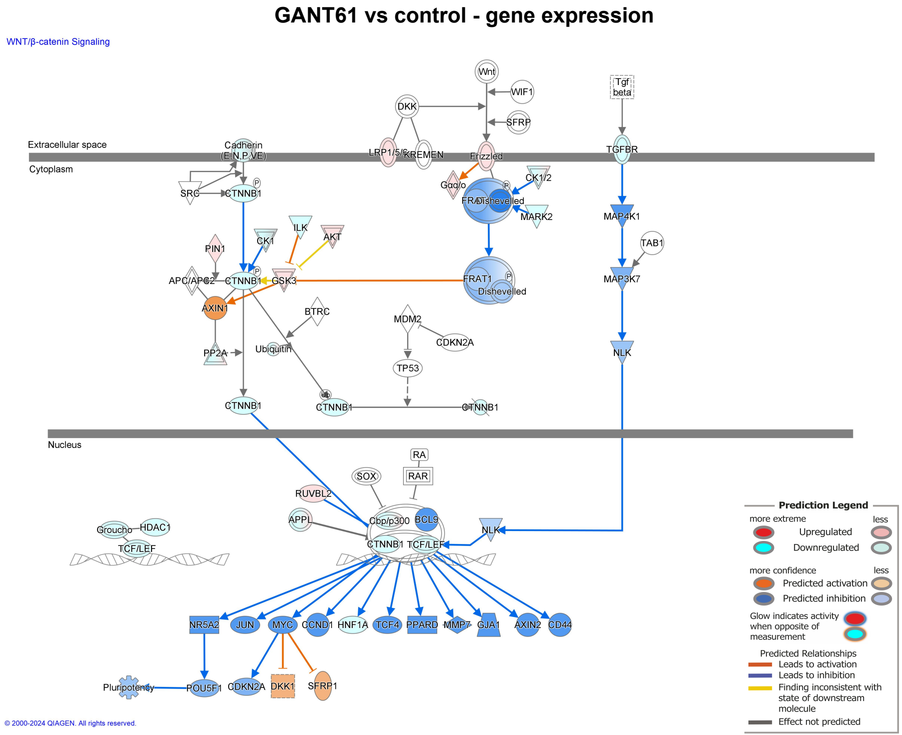

Supplement Figure 5 - interactive pdf:

Wnt signaling pathway and its detailed dynamics during culture of primary hepatocytes with Hh modulators. The heatmap shows the activation Z-score analysis based on RNA-Seq. (top) and proteomics (down) of male hepatocytes incubated with the Hh modulators compared to the control, respectively. The activation Z-score was calculated with IPA software. The p-value cutoff of 0.05 was used for calculation. A click on the colored squares reveals the detailed pathway analysis of the Wnt pathway of compound incubation compared to control incubation after 48 h done by IPA. Nodes with color gradients represent complexes whose individual components are regulated differently. Lines symbolize direct interaction. Dashed lines symbolize indirect interaction.

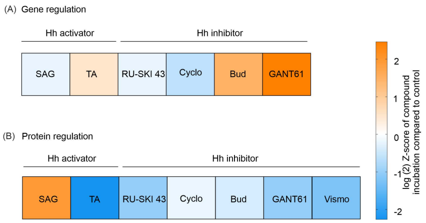

SAG vs control - protein translation

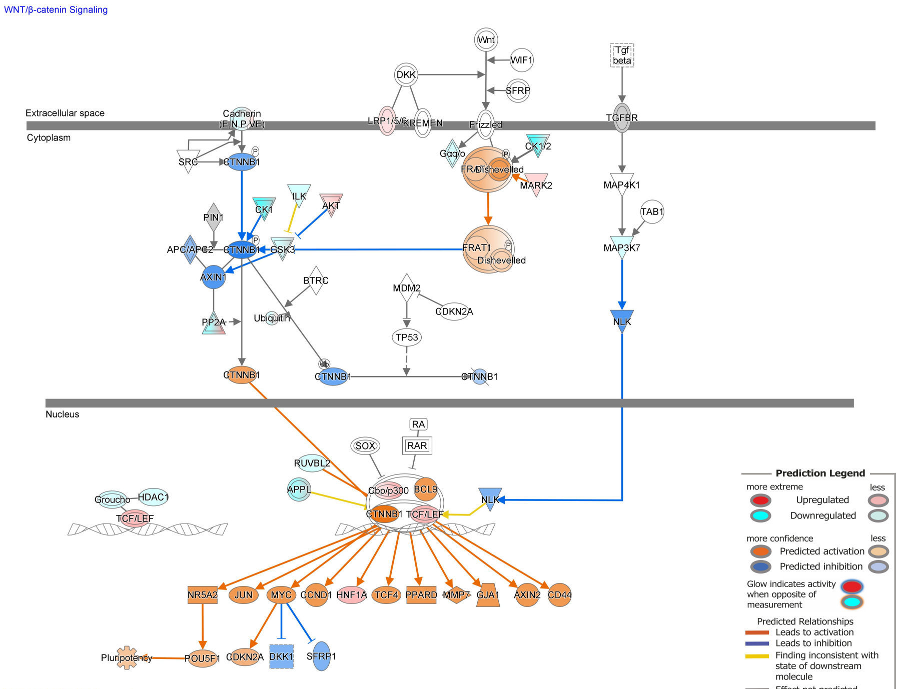

© 2000-2024 QIAGEN. All rights reserved.

Supplement Figure 5 - interactive pdf:

Wnt signaling pathway and its detailed dynamics during culture of primary hepatocytes with Hh modulators. The heatmap shows the activation Z-score analysis based on RNA-Seq. (top) and proteomics (down) of male hepatocytes incubated with the Hh modulators compared to the control, respectively. The activation Z-score was calculated with IPA software. The p-value cutoff of 0.05 was used for calculation. A click on the colored squares reveals the detailed pathway analysis of the Wnt pathway of compound incubation compared to control incubation after 48 h done by IPA. Nodes with color gradients represent complexes whose individual components are regulated differently. Lines symbolize direct interaction. Dashed lines symbolize indirect interaction.

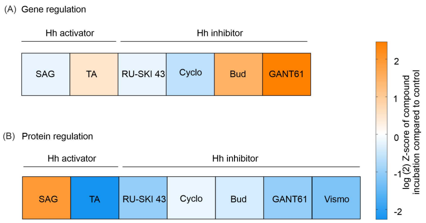

TA vs control - protein translation

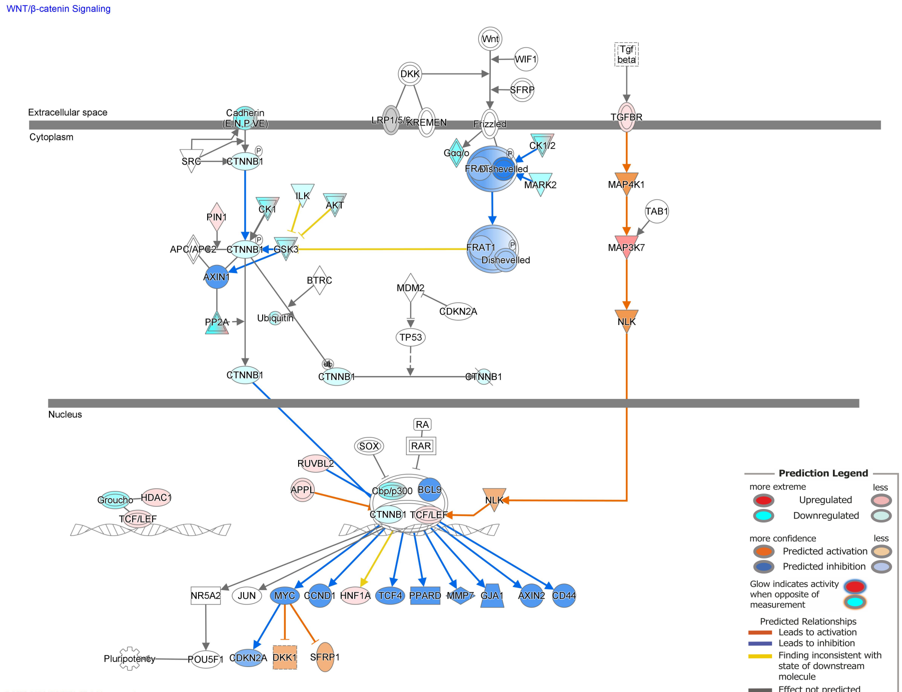

© 2000-2024 QIAGEN. All rights reserved.

Supplement Figure 5 - interactive pdf:

Wnt signaling pathway and its detailed dynamics during culture of primary hepatocytes with Hh modulators. The heatmap shows the activation Z-score analysis based on RNA-Seq. (top) and proteomics (down) of male hepatocytes incubated with the Hh modulators compared to the control, respectively. The activation Z-score was calculated with IPA software. The p-value cutoff of 0.05 was used for calculation. A click on the colored squares reveals the detailed pathway analysis of the Wnt pathway of compound incubation compared to control incubation after 48 h done by IPA. Nodes with color gradients represent complexes whose individual components are regulated differently. Lines symbolize direct interaction. Dashed lines symbolize indirect interaction.

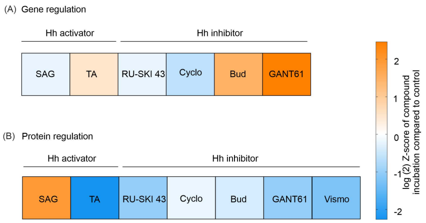

RU-SKI 43 vs control - protein translation

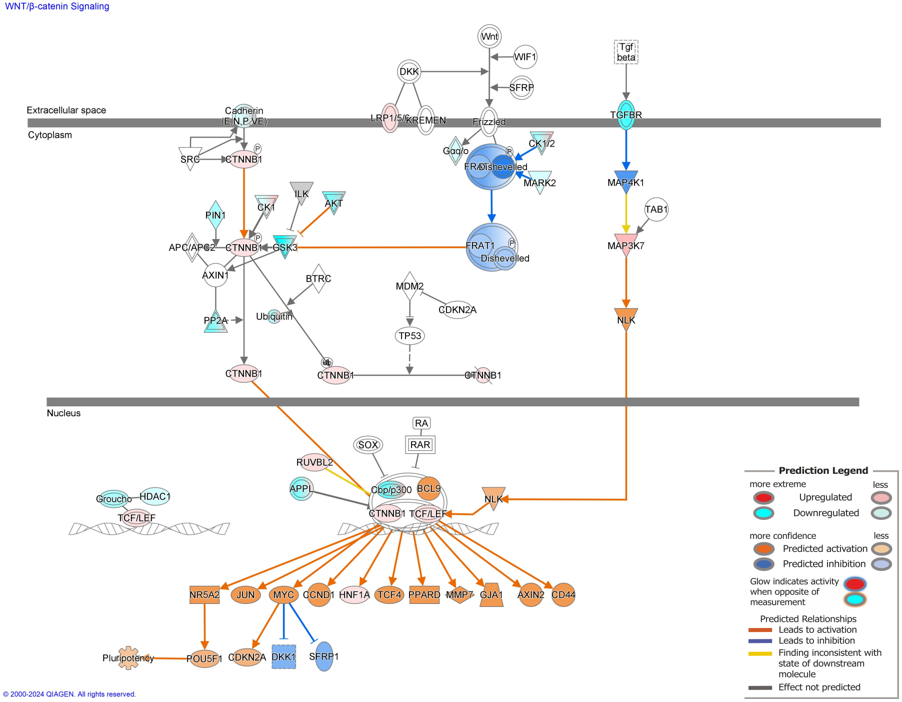

Supplement Figure 5 - interactive pdf:

Wnt signaling pathway and its detailed dynamics during culture of primary hepatocytes with Hh modulators. The heatmap shows the activation Z-score analysis based on RNA-Seq. (top) and proteomics (down) of male hepatocytes incubated with the Hh modulators compared to the control, respectively. The activation Z-score was calculated with IPA software. The p-value cutoff of 0.05 was used for calculation. A click on the colored squares reveals the detailed pathway analysis of the Wnt pathway of compound incubation compared to control incubation after 48 h done by IPA. Nodes with color gradients represent complexes whose individual components are regulated differently. Lines symbolize direct interaction. Dashed lines symbolize indirect interaction.

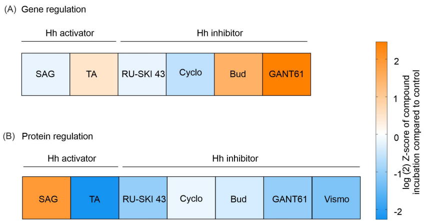

Cyclo vs control - protein translation

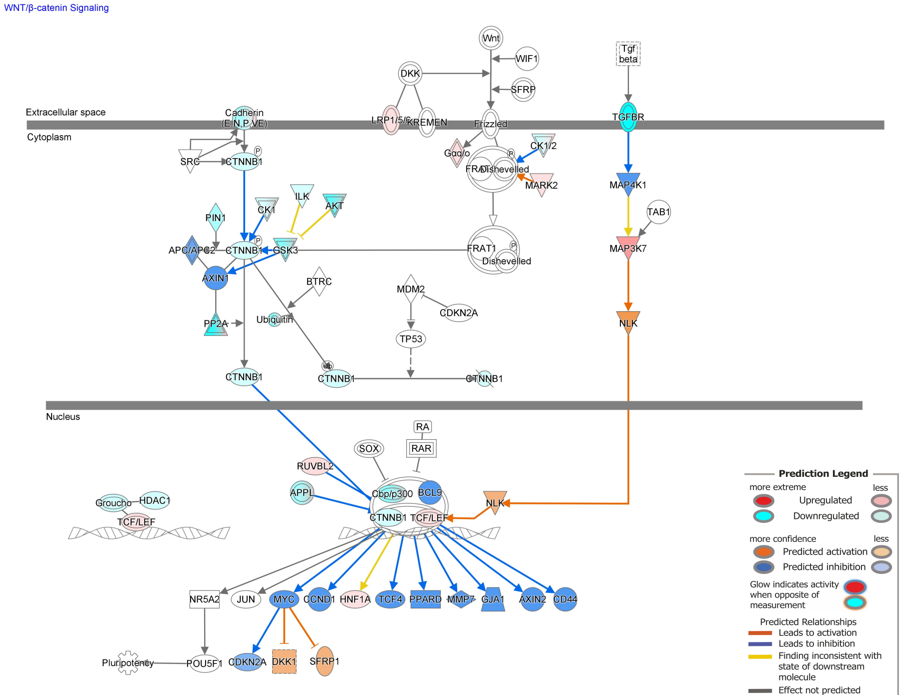

© 2000-2024 QIAGEN. All rights reserved.

Supplement Figure 5 - interactive pdf:

Wnt signaling pathway and its detailed dynamics during culture of primary hepatocytes with Hh modulators. The heatmap shows the activation Z-score analysis based on RNA-Seq. (top) and proteomics (down) of male hepatocytes incubated with the Hh modulators compared to the control, respectively. The activation Z-score was calculated with IPA software. The p-value cutoff of 0.05 was used for calculation. A click on the colored squares reveals the detailed pathway analysis of the Wnt pathway of compound incubation compared to control incubation after 48 h done by IPA. Nodes with color gradients represent complexes whose individual components are regulated differently. Lines symbolize direct interaction. Dashed lines symbolize indirect interaction.

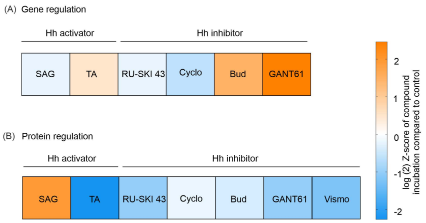

Bud vs control - protein translation

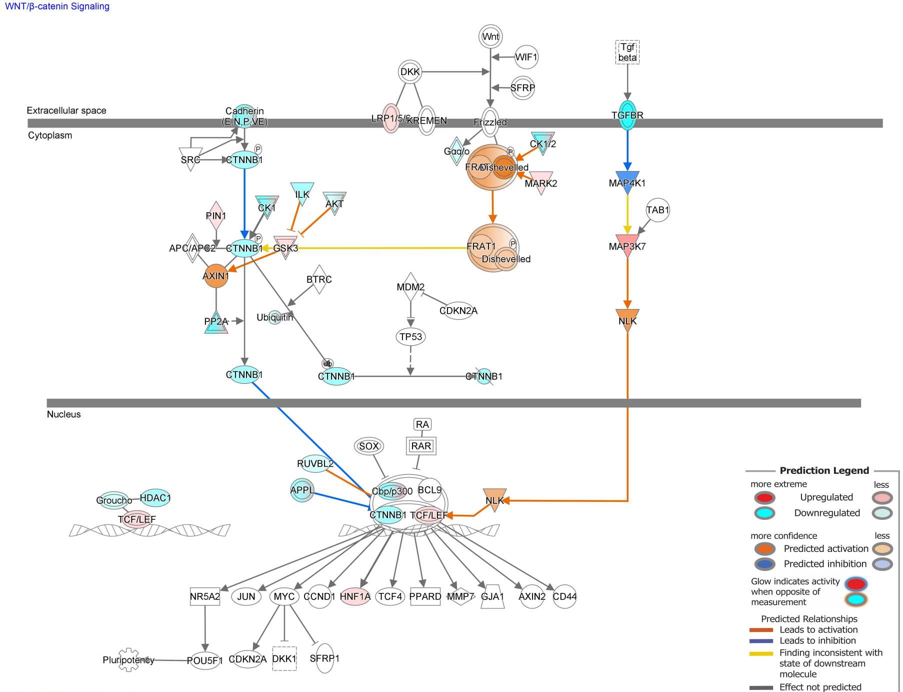

© 2000-2024 QIAGEN. All rights reserved.

Supplement Figure 5 - interactive pdf:

Wnt signaling pathway and its detailed dynamics during culture of primary hepatocytes with Hh modulators. The heatmap shows the activation Z-score analysis based on RNA-Seq. (top) and proteomics (down) of male hepatocytes incubated with the Hh modulators compared to the control, respectively. The activation Z-score was calculated with IPA software. The p-value cutoff of 0.05 was used for calculation. A click on the colored squares reveals the detailed pathway analysis of the Wnt pathway of compound incubation compared to control incubation after 48 h done by IPA. Nodes with color gradients represent complexes whose individual components are regulated differently. Lines symbolize direct interaction. Dashed lines symbolize indirect interaction.

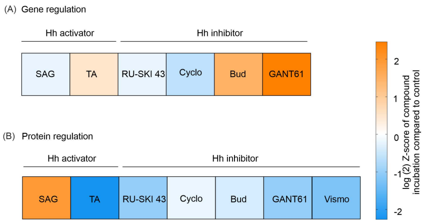

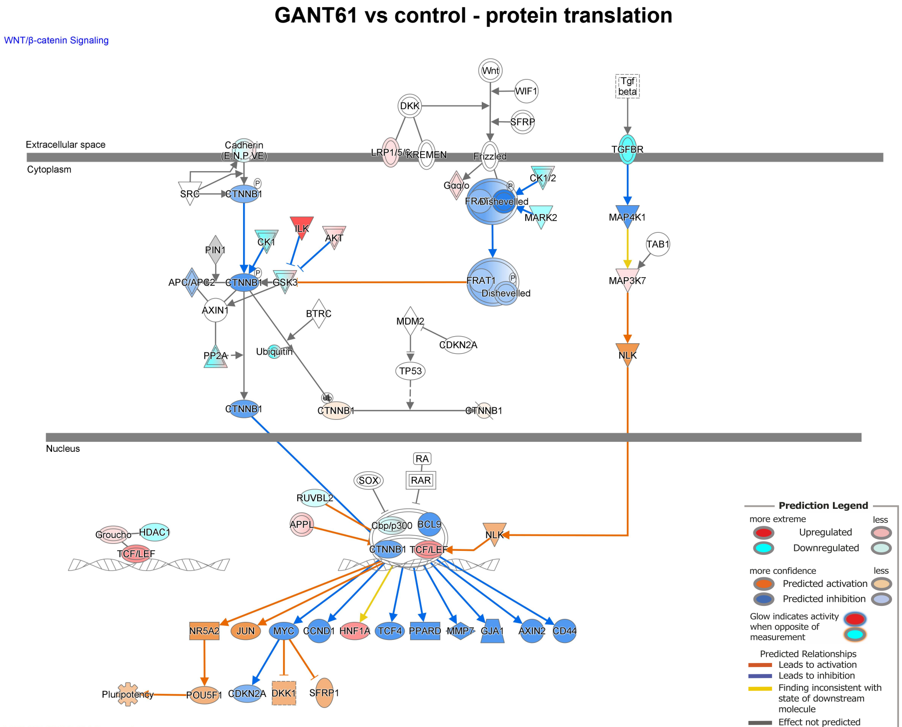

© 2000-2024 QIAGEN. All rights reserved.

Supplement Figure 5 - interactive pdf:

Wnt signaling pathway and its detailed dynamics during culture of primary hepatocytes with Hh modulators. The heatmap shows the activation Z-score analysis based on RNA-Seq. (top) and proteomics (down) of male hepatocytes incubated with the Hh modulators compared to the control, respectively. The activation Z-score was calculated with IPA software. The p-value cutoff of 0.05 was used for calculation. A click on the colored squares reveals the detailed pathway analysis of the Wnt pathway of compound incubation compared to control incubation after 48 h done by IPA. Nodes with color gradients represent complexes whose individual components are regulated differently. Lines symbolize direct interaction. Dashed lines symbolize indirect interaction.

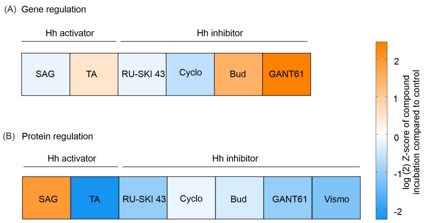

Vismo vs control - protein translation

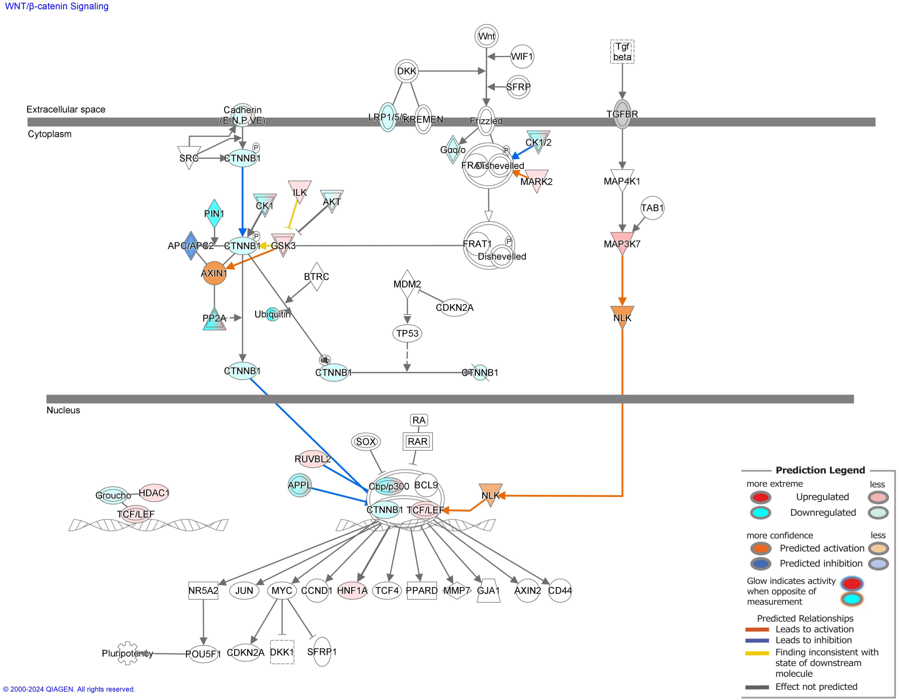

Supplement Figure 5 - interactive pdf:

Wnt signaling pathway and its detailed dynamics during culture of primary hepatocytes with Hh modulators. The heatmap shows the activation Z-score analysis based on RNA-Seq. (top) and proteomics (down) of male hepatocytes incubated with the Hh modulators compared to the control, respectively. The activation Z-score was calculated with IPA software. The p-value cutoff of 0.05 was used for calculation. A click on the colored squares reveals the detailed pathway analysis of the Wnt pathway of compound incubation compared to control incubation after 48 h done by IPA. Nodes with color gradients represent complexes whose individual components are regulated differently. Lines symbolize direct interaction. Dashed lines symbolize indirect interaction.
